# Enhancing Vacuum Membrane Distillation Performance via Asymmetric NACA 2412 Airfoil-Based Mixing Promoters

**DOI:** 10.3390/membranes16100313

**Published:** 2026-09-24

**Authors:** Umar F. Alqsair

**Affiliations:** Department of Mechanical Engineering, College of Engineering, Qassim University, Buraidah 51452, Saudi Arabia; u.alqsair@qu.edu.sa

**Keywords:** vacuum membrane distillation (VMD), NACA 2412 airfoil, boundary-layer polarization, computational fluid dynamics (CFD), permeate flux enhancement, hydrodynamic optimization

## Abstract

Even though vacuum membrane distillation (*VMD*) for low-temperature desalination is an innovative technology, industrial applications remain limited by the concentration and thermal polarization, and conventional passive spacers mitigate these issues at the cost of high hydraulic energy consumption. This article aims to reduce boundary-layer limitations using an asymmetric NACA 2412 cambered airfoil that acts as a passive mixer. The effect of the airfoil’s angle of attack (*AoA*) was assessed in both upward and downward orientations, with airflow modeled at Reynolds numbers of *Re* = 1000 and 1500. The results show that the airfoil in the upward orientation was highly effective at directing airflow inducing flow separation and coherent wake structures within the boundary layer, while maintaining low form drag and a correspondingly small hydraulic penalty. The upward design at *AoA* = 12° improved the permeate flux by 14.6% compared to the zero-incidence baseline (*AoA* = 0°) at *Re* = 1500, reaching a permeate flux of 66.03 kg/m^2^h. This gain came at a modest hydraulic cost, with the channel pressure drop rising by only ≈10 Pa (from 13.33 to 23.52 Pa). These results indicate that the airfoil-based mixing promoter offers a favorable balance between flux enhancement and the hydraulic penalty, making it a promising passive design strategy for *VMD* channels.

## 1. Introduction

The global shortage of fresh water has created greater interest in more efficient, sustainable solutions for desalination and water purification. As a result, one of the most promising new technologies for membrane-based separation is vacuum membrane distillation (*VMD*). *VMD* can operate at much lower feed temperatures than traditional thermal distillation because a vacuum is maintained on the permeate side, creating a vapor pressure gradient across the hydrophobic membrane. This results in several thermodynamic advantages over traditional thermal distillation methods, including the virtually 100% rejection of non-volatile solutes, lower sensible heat losses, and the potential to use low-grade industrial waste heat or solar thermal energy as a feed source for the distillation process [1,2,3,4,5].

The global desalination industry currently produces ≈95.37 million m^3^/day of potable water, with the vast majority produced via membrane-based Reverse Osmosis (*RO*), which accounts for 68.7% of the installed capacity. In contrast, multi-stage flash (MSF) and multi-effect distillation (MED) account for 17.6% and 6.9%, respectively [6]. Within the family of thermally driven, heat-requiring technologies such as *MSF* and *MED*, membrane distillation (*MD*) is a commercially underdeveloped yet strategically important technology that requires no hydraulic pressure and operates at a much lower feedwater temperature than traditional technologies. Furthermore, because *MD* processes are largely insensitive to variations in feedwater salinity, the technology is an excellent choice for co-generating potable water from either solar–thermal or waste thermal energy sources [6]. These distinguishing energy input requirements of the *MD* technologies become apparent when contrasting the average performance values tabulated by Curto et al. [6] *MSF* requires 190–390 kJ/kg thermal and 4–6 kWh/m^3^ electric energy and yields a product cost of $0.56–1.75/m^3^, *MED* requires 230–390 kJ/kg thermal and 1.5–2.5 kWh/m^3^ electric and costs $0.52–1.50/m^3^, whilst *SWRO* is driven solely by electric energy (3–6 kWh/m^3^) costs ranging from $0.45 to $1.72/m^3^. By leveraging the waste thermal energy already being rejected from some industrial processes, *MD*’s energy input cost is minimal when operating in cogeneration mode or as a waste-heat process. Desalination in the MENA (Middle East and North Africa) region accounts for almost half (47.5%) of global capacity, with high seawater salinity and the availability of cheap thermal energy from co-located industrial plants providing ideal conditions for *MD* utilization. Among the different operating *MD* configurations, the direct contact (*DCMD*), air gap (*AGMD*), sweeping gas (*SGMD*), and vacuum (*VMD*) types, the *VMD* configuration generates the highest thermodynamic driving force because a vacuum is applied on the permeate side, thereby removing the resistance from the condensation side in other forms of *MD*. Exergy analysis of different *MD* families shows that *DCMD* has a thermodynamic efficiency of less than 1%. In contrast, *RO* has an efficiency of about 31.9%. In contrast, *DCMD* loses energy due to boundary-layer effects, flow losses on the feed side of the module, and overall heat-transfer resistance within the module. This underscores the need to minimize internal irreversibility in the feed channel chiefly polarization phenomena to maximize the process driving force.

Although its theoretical potential is immense, the practical *VMD* performance has long been limited by two closely intertwined boundary-layer phenomena that erode this driving force: temperature polarization (*TP*) and concentration polarization (*CP*). While water vapor passing across a membrane results in a localized decrease in temperature on the feed side (owing to latent heat consumed, also represented by the temperature polarization coefficient (*TPC*)), non-volatile solutes are rejected and concentrated at the feed-membrane boundary (also described by the concentration polarization coefficient (*CPC*)). Both *TPC* and *CPC* sharply reduce the driving pressure gradient for vapor flow, worsen membrane fouling, and significantly reduce transmembrane permeate flux [7,8,9]. To minimize these boundary effects, hydrodynamic optimization with passive mixing elements has proved quite effective [10,11,12]. Conventional methods for promoting mixing employ spacer nets or static obstacles placed in the feed-membrane channels, which lead to high hydraulic losses and penalize the pumping energy consumption. National Advisory Committee for Aeronautics (NACA) airfoils can achieve high localized vorticity and controllable flow separation with a minimal form-drag penalty, owing to their inherent design features and an optimized lift-to-drag ratio; such characteristics are extremely important for generating intense, chaotic advection within the limited volume of membrane channels. Prior work has concentrated on symmetric airfoil profiles or flat-plate inserts; to the author’s knowledge, no study has systematically examined asymmetric cambered airfoils oriented either upward or downward as passive mixers for *VMD*. The influence of the *AoA* under turbulent flow conditions in a membrane process, specifically in the *VMD* application, has not been extensively analyzed to date. Very few studies have examined how flow field modification through airfoils could reduce local *TPC* and *CPC*, thereby inducing a considerable net flux increase with low power consumption. To overcome these obstacles and make progress towards advanced high-performance *VMD* membrane modules, this study carried out a two-dimensional CFD examination of a *VMD* feed channel containing a 5 mm chord length sub-macroscale NACA 2412 airfoil as a passive mixer at three angles of attack (*AoA*) = 5°, 8°, and 12° in two Reynolds numbers, 1000 and 1500, in the turbulent regime using the *k* − *ω SST* turbulence model. For Reynolds number (*Re* = 1000 and 1500), a baseline case is analyzed at a zero angle of attack (*α* = 0°). We investigate and compare the *TPC*, *CPC*, and average transmembrane flux (*J*) across all these cases. This work aims to achieve significant improvements in *VMD* performance by rationally designing channel hydrodynamics based on aerodynamic profiles.

## 2. Literature Review

### 2.1. Fundamental Principles and Transport Mechanisms of Membrane Distillation

Membrane distillation (*MD*) uses vapor separated from the liquid across a microporous hydrophobic membrane, driven by a vapor pressure gradient generated by a temperature difference. The concept and first theory are well summarized by Lawson and Lloyd [1], who address module design, membrane characteristics, coupled heat and mass transfer, and a lack of understanding for non-boiling temperatures. The membrane pore size, porosity, tortuosity, thickness, and liquid entry pressure interact with operating conditions across the four configurations, as is seen in Alkhudhiri et al. [2], who conclude that selection cannot be viewed independently from the feed channel. *VMD* was detailed by Abu-Zeid et al. [3] in their review of the method’s various transport models, membranes, and applications, noting its potential for use with low-grade heat if system performance can offset losses in concentration and energy consumption. More specifically, regarding *VMD* transport across various configurations and potential applications, Chiam and Sarbatly [4] classify problems as single, binary, or multicomponent transport and suggest that the membrane permeability coefficient is the simplest selection criterion. The total compiled experimental performance data, presented in Alklaibi and Lior [5] spanning *MD* applications over several generations, demonstrate fluxes from 2.8 to 129 kg/m^2^h, with feed temperature as the most effective design lever (a gain in flux of more than 3 fold between 50–70 °C), concluding that enhanced performance requires the elimination of Near-Membrane Mass/Heat Transport. Most of the work dealing with the transport models and mechanisms for *VMD*, both experimental and theoretical, has come from the laboratory of Bandini-Sarti. Their first seminal paper, Bandini et al. [13], demonstrated that the total resistance to vapor flux consisted of two parts: one due to heat and mass transfer in the liquid, and a second due to mass transfer through the pore; depending on the membrane, one of these dominates. A model developed for transporting dilute aqueous organics led Bandini et al. [7] to conclude that the vapor-phase pressure (i.e., the downstream vacuum pressure) is the more important design variable. While concentration polarization has been modeled, its strong influence on selectivity has not yet been experimentally determined. Their approach for studying aroma losses due to concentration polarization in fruit juices, when applied to the grape juice concentration, experimentally demonstrated that polarization, not permeability, was responsible for a 90% loss of aromatics when grapes must be concentrated to near-sugar concentrations. A successful multicomponent model was presented by Soni et al. [8] for the recovery of aroma compounds from black currant juice with the UNIFAC (Universal Quasichemical Functional-group Activity Coefficients) group-contribution method for thermodynamical interaction and Knudsen/viscous transition region for gas diffusion inside membrane pores and showed sensitivity to the vapor and activity coefficients of different components. Al-Asheh et al. [9] have explored the effect of increasing the sucrose solution concentration with varying channel lengths, showing that the benefit in flux diminishes at a certain level, apparently due to the decreasing total thickness of the boundary layer along the channel and indicating that most benefit is achieved within the laminar region of flow. Regarding the mechanisms of the mass transport of vapor and gas through a porous medium, Kast and Hohenthanner [10] have devised an analytical formula combining the molecular and viscous flow regimes; this work has been extended by Ding et al. [11] to a *DCMD* model where the Poiseuille regime of gas flow becomes important under high-temperature feed conditions. Among *VMD* applications, methods of analysis and experimental correlations are compiled by Baghel et al. [12], who lament that this flux advantage is quickly lost to polarization and fouling. Bandini and Sarti [14] published what is believed to be the simplest method for testing whether the liquid-side mass-transfer (SMT) membrane more strongly controls vapor transmission. Dao et al. [15] published a new steady-state temperature-increase method that is more stable and better suited for testing membrane vapor permeability. A *PVDF* homogeneous and heterogeneous hollow-fiber model, based on the Knudsen–viscous combined transport model, was presented by Liu et al. [16], which concluded that, among the various membrane properties, pore size is the most sensitive variable for *VMD* flux performance. To show a comparison of pervaporation (*PV*) and *VMD* performances on the same *PVDF* membranes and module, Khayet and Matsuura [17] developed a *VMD* pore-size-distribution-resolved model in which the solution-diffusion contributions for a dense matrix (around 30% at 10 µm) decreased for membranes larger than 5 µm. At the same time, liquid boundary-layer mass transfer becomes the most important limiting factor for membranes with higher permeation characteristics. Eykens et al. [18] experimentally compared four membrane distillation configurations (*DCMD*, *AGMD*, *PGMD*, *VMD*) under identical driving-force conditions using a factorial Design of Experiments to assess the effects of temperature, flow velocity, salinity, and membrane type. They found that the flux order was *VMD* > *DCMD* > *PGMD*0 > *PGMD*2 > *AGMD*, with configuration choice having a greater impact on flux than membrane selection.

### 2.2. Polarization Phenomena: Quantification, Modeling, and Mitigation

Temperature and concentration polarization are the primary performance-loss mechanisms in *MD*, and numerous experimental studies have quantified them across different *MD* configurations and feed waters. Martínez-Dez and Vázquez-González [19] measured water permeation rates across flat *PTFE* membranes with *NaCl* solutions and formulated a generalized vapor-pressure-polarization coefficient, demonstrating the increasing economic cost of concentration polarization with increasing salinity. Gryta and Tomaszewska [20] developed improved interfacial temperature equations for capillary-driven *DCMD* modules that match experimental data to within 6% and indicated that only Nusselt correlations scaled to the appropriate geometric parameters accurately predict the module’s heat-transfer coefficients. A parametric *VMD* investigation by Banat and Simandl [21], using normalizing sensitivity analysis factors, proved that the operation involves two serial resistances in series and that overcoming the heat-transfer limitations is achieved by reducing the membrane module heat-transfer resistance; an investigation of *VMD* of sucrose solutions by Al-Asheh et al. [9] found that flow-rate gains diminish at very high fluxes and for the most concentrated solutions and concluded that reduced water activity plus high polarization resulted in the observed decreases in water flux as the solute concentration increases. Taguchi optimization techniques applied to a hollow-fiber *VMD* apparatus by Alsalhy et al. [22] yielded a performance of ≈66 kg/m^2^h with the vacuum pressure and feed temperature being the significant parameters, 66% variability. The first direct measurements of a long-term air-gap *MD* operation with 1500 h tests on *PVDF* and *PTFE* membranes showed stable water flux, high salt rejection (≈99%), little flux loss with an increasing *NaCl* concentration up to 10 wt.% (6% loss), and no detectable thermal diffusion effect.

The relationship among membrane polarization, surface characteristics, module construction, and the potential for fouling has been established in several studies. By comparing experimental data with CFD results for a superhydrophobic *PVDF* membrane (162° contact angle), Liu et al. [23] observed surface-induced slip that enhanced flow near the membrane and significantly reduced concentration polarization relative to temperature polarization. Julian et al. [24] compared 2D CFD predictions of fouling in a *VMD* crystallization cell and found that neglecting temperature polarization led to a 40% overestimation of CaCO_3_ fouling, whereas neglecting concentration polarization resulted in only a 23% underprediction, thereby underscoring the need to include both effects. Studies on asymmetric hollow fibers by Zhang et al. [25] revealed a more than 4-*fold* increase in flux when the dense skin layer was oriented towards the hot feed stream, which was further increased by applying a vacuum on the permeate side. Very high fluxes (>71 kg/h) were obtained using coated polypropylene crossflow fibers by Li et al. [26] in *VMD* operation, demonstrating good temperature polarization coefficient performance (*TPCs* = 0.93–0.99) and highly enhanced crossflow heat transfer coefficients (*CFHTCs* > 4 vs. crossflow HTCs for parallel flow at the same *Re*).

CFD is an established tool to analyze polarization profiles across *MD* modules. Tang et al. [27] successfully modeled a tubular *MD* module by considering the membrane as a continuum porous medium and replicating measured profiles for both water flux and temperature, while Upadhyaya et al. [28] modeled a planar channel flat *MD* device and performed experimental validation of the model by introducing a temperature-dependent vapor-water properties equation of the Antoine form. Bouguecha et al. [29] used a 3D CFD code to visualize streamlines and temperature distribution in a renewable-energy-driven *VMD* pilot plant. Baghel et al. [30] developed a 3D simulation of an industrial *VMD* plant dealing with textile waste streams; interfacial temperatures were significantly lower than those in the bulk solution, confirming temperature polarization. Chernyshov et al. [31] successfully integrated momentum, heat, and species transport in an *AGMD* setup and indicated a relationship between near-membrane polarization and potential fouling. Further experimental support came from studies by Xu et al. [32,33] on a pilot-scale *MD* module, which confirmed decreased temperature polarization and increased flux with rising feed Reynolds numbers, and whose computational results were corroborated to a considerable extent via a FLUENT simulation. Yadav et al. [34] quantified the benefits of raising the flow-rate Reynolds number of an *AGMD* from 50 to 200, which provided only a 13% increase in flux. In addition, increasing the feed-water temperature resulted in a 132.9% improvement. By integrating their *MD* model with a Genetic Algorithm (*GA*), Zuo et al. [35] achieved a 38% cost reduction, while the models of hollow-fiber *PVDF*-based *MD* (for treating oil/water mixture and volatile organic compounds (*VOCs*) and the separation of *VOCs* via a hollow-fiber *MD*, as developed by Tahvildari et al. [36] and Ghadiri et al. [37], accurately predict the entire coupled fields. Alsaadi et al. [38] conducted an experimental and theoretical modeling study on temperature polarization in vacuum membrane distillation (*VMD*), validating a mathematical model against flux data at varying temperatures and pressures. They found that the temperature polarization coefficient (*TPC*) decreased with an increasing feed temperature and decreasing permeate pressure and that integrating heat recovery for scale-up worsened polarization effects. Efome et al. [39] experimentally fabricated *PVDF*/*SiO*2 nanocomposite membranes via phase inversion and tested their vacuum membrane distillation (*VMD*) performance at varying nanoparticle concentrations (1–10 wt.%). They found an optimal loading of 7 wt.%, achieving a 4-*fold* increase in flux (0.7 to 2.9 kg/m^2^h) with salt rejection above 99.98%. Garofalo et al. [40] conducted an experimental study to scale up *MFI* zeolite membranes (30 cm long) prepared via secondary growth with cross-flow seeding, testing different seeding rotation times and evaluating their desalination performance in vacuum membrane distillation (*VMD*) using deionized water and *NaCl* solutions. They characterized the membranes using *SEM*, *XRD*, *EDX*, and gas permeation, finding that the membrane with a 72 min seeding time performed best, achieving permeate fluxes of 13.8 kg/m^2^h and 8.2 kg/m^2^h with rejections of 99.8% and 99.6% for 0.2 M and 0.9 M feeds, respectively, with performance fully restorable via cleaning. Liu et al. [41] design an experimental study using an icing wind tunnel to investigate saltwater droplet ice accretion on an NACA0018 airfoil. They examined the effects of temperature, *LWC*, *MVD*, salinity, and angle of attack on the ice thickness and coverage area. They found that higher salinity reduces the ice thickness/area (up to ≈35% at high *LWC*), while lower temperature, higher *LWC*, and larger *MVD* increase ice accretion and amplify salinity’s inhibiting effect.

### 2.3. Passive Mixing Promoters and Novel Channel Geometries

Passive inserts are the most extensively investigated alternative for disrupting boundary layers in line with active flow control. Bouguecha et al. [29] have solved the coupled vorticity and energy equations for several types of feed–cavity configurations, demonstrating that the overall flow pattern shapes the relationship between convective circulation and transmembrane transfer. Based on a *DCMD* simulation with the insertion of a spacer, El Kadi and Janajreh [42] computed that inserting a conductivity spacer can improve the temperature polarization coefficient (*TPC*) by up to 50% and the total flux by 35% and concluded that concentration polarization alone causes about 10% loss of flux. In a 3D CFD simulation, Hasani et al. [43] noted that 2D simulations cannot capture adjacent dormant zones and the associated circulation streams near filaments. They stated that staggered configurations yield the most homogeneous polarization distribution. Through experiments on *DCMD* in a tubular module equipped with a helical strake that generates secondary helical vortices, a flux gain of up to 44% was achieved by Al-Muallem et al. [44]. Loussif and Orfi [45] numerically analyzed the insertion of metal foams into *VMD*, which increased the flux up to threefold in low-flow regimes. In contrast, at *Re* = 1500, the increased cost of the fluid resulted in a pressure drop of 3.33 kPa for the metallic insert, compared to 0.362 kPa for the unfitted tube. A *DCMD* process with recirculation dead zones can be effectively boosted to 134.7% in flux by removing only the dead zones, as reported by Khatab et al. [46]. Alwatban et al. [47] analyzed the effect of net-type spacers in a 2D *VMD* module with feed temperatures of 40–80 °C for *Re* of 400–1500. They determined that the largest spacer (0.5 h) increased flux by up to 53% at *Re* = 1500 and *T_feed_* = 80 °C. In contrast, the periodic renewal of the feed boundary layers, resulting from their configuration, is suggested by the characteristics of the polarization profiles observed in local waveform patterns. A double-suction configuration including a centrally perforated tube at the module scale helps to reclaim about 50–70% of the flux decrease caused by the unfixed shell-side feed vacuum distribution and increases the specific energy saving by 12.5% Pang et al. [48]. and Seo et al. [49] used a validated CFD model to compare 51 *DCMD* spacer designs, finding that a symmetric circular–zigzag configuration maximized vapor flux (26% above that of an empty channel). They recommend larger, denser spacer arrays when maximizing flux is the priority and smaller, sparser ones when minimizing pressure drop is the priority. Tan et al. [50] experimentally and computationally compared metallic (*Ni*, *Cu*) spacers against polypropylene in *DCMD*. Metallic foam spacers reduce heater energy input per unit distillate by up to 16%, with platinum-coated Ni foam cutting it by 28% under light irradiation. Ni et al. [51] used 3D CFD to compare curved and straight spacers in *DCMD*. Curved spacers improved mixing and permeated flux at a higher pressure-drop cost, with the optimal geometry shifting with the Reynolds number. Thomas et al. [52] experimentally coated 3D-printed *DCMD* spacers with fluorinated silica nanoparticles to reduce scaling. The coating cut scalant attachment by 74% and membrane surface scaling by 60%, via increased hydrophobicity and reduced surface energy. Albeirutty et al. [53] experimentally tested 3D-printed spacers with varying attack and filament angles in MD channels. A 45° attack angle with a 90° filament angle gave the highest heat transfer, while filament angles above 90° resulted in a higher pressure drop. Jeong et al. [54] combined experiments and mechanistic modeling to explain how a 3D-printed *CNT* spacer mitigates *DCMD* scaling. The spacer’s nanoscale roughness strengthened hydrogen bonding, delaying crystallization and forming larger, less-adherent crystals. Al-juboori et al. [55] experimentally and theoretically tested low-power ultrasound delivered via metallic spacers in *AGMD*. Flux improved by up to 400%, mainly through enhanced membrane permeability, with no observed membrane damage. Ahmed et al. [56] experimentally used *MgO*-coated *Ni* − *Cr* spacers for localized Joule heating in *DCMD*. A periodic 0.2 A/cm^2^ current increase permeates flux by 15% while increasing energy consumption by only 4%, without compromising membrane properties. Jeong et al. [57] used CFD and 3D-printed prototypes to test a helical strip-type spacer against conventional double-layer spacers in *MD*. The helical design boosted water flux by up to 27% (20% experimentally) through multi-directional mixing, at the cost of a pressure drop up to 1.9 times higher. Jeong et al. [58] combined experiments and CFD to test 3D-printed carbon nanofiller-embedded spacers with engineered multi-scale roughness. The 2% *CNT* spacer increased permeate flux by 75% relative to the *PLA* control while also reducing ion permeation, thereby mitigating both *CP* and *TP*. Ahmed et al. [59] used 2D CFD to compare conductive (copper) and non-conductive (polymer) cylindrical spacers in *DCMD*. Copper outperformed the polymer, but neither beat the no-spacer baseline, as spacer-induced recirculation created stagnant zones that degraded flux and thermal efficiency at medium-to-high Reynolds numbers. Li et al. [60] combined experiments and CFD to test a 3D-printed helical baffle, superhydrophobic coating, and intermittent operation in vacuum *MD*. The baffle boosted flux by 6–46% with minimal additional pumping power, and combining all three features yielded over 6% higher flux and over 100% greater durability than the baseline. Chang et al. [61] developed a 3D CFD model of spacer-filled *DCMD* channels capturing transmembrane heat and mass transfer along the full module length. They developed friction factors and Nusselt number correlations showing spacer-induced heat transfer enhancement depends on both the spacer geometry and Reynolds number. Karimi et al. [62] developed a validated CFD model to screen novel *DCMD* spacer geometries (twisted, elliptical, pillar, multilayer). Their twisted and elliptical designs achieved up to 5.4% lower temperature polarization than the best literature spacers, with the twisted spacer giving the highest flux and the elliptical design better limiting concentration polarization. Kim et al. [63] combined experimental and theoretical modeling to study nonwoven net spacers on the feed and/or permeate sides of *DCMD*. Feed-side spacers gave larger flux gains (21–33%) than permeate-side spacers (7–19%), with the thinnest, densest 90° spacer on both sides yielding a maximum 43% flux enhancement. Alwatban et al. [64] used CFD to simulate *DCMD* with net-type spacers embedded into the membrane surface, creating a micro-patterned surface. This significantly mitigated concentration and temperature polarization, boosting water flux by about 40% with the larger-strand design. Hagedorn et al. [65] experimentally evaluated commercial membranes and spacers for full-scale *MD* design, measuring the pressure drop, heat transfer, *LEP*, and flux. Spacers with a high mesh-size-to-thickness ratio gave the best pressure drop performance, while high pore-size-to-thickness membranes yielded the highest flux. Zhang et al. [66] used 2D CFD to test semicircular spacers in micro-*VMD* modules. The spacers improved the transmembrane mass flow rate and the temperature polarization coefficient, with performance more sensitive to the feed temperature than to feed velocity. Alshwairekh et al. [67] developed a CFD study (ANSYS, *k* − *ω SST* turbulence models) on membrane and channel corrugations in forward osmosis modules. They compared to single, double, and combined-membrane/channel corrugations across *Re* = 300–1500 using a modified solution-diffusion flux model. They found that single corrugation best mitigated concentration polarization, while combined corrugation gave ≈15% higher water flux; single/double corrugation at *Re* = 300 had the best overall coefficient of performance. Zhou and Ladner [68] developed a CFD study using COMSOL on discrete-object feed spacers 3D-printed directly onto *RO* membranes. They optimized the spacer height, shape (ellipses vs. airfoils), and array spacing to minimize the pressure drop and concentration polarization. They found that elliptical spacers (0.42 mm height) matched conventional spacer pressure drop while enabling a 43% higher membrane packing density and 7% CP reduction.

### 2.4. Enhanced Configurations, Fouling, and Wastewater Applications

Naidu et al. [69] carried out an experimental and modeling study on vacuum-enhanced direct contact membrane distillation (*V* − *DCMD*) for treating *RO* concentrate wastewater, examining transport mechanisms, temperature profiles, and fouling. They found that *V* − *DCMD* achieved 37.6% higher flux than *DCMD* at the same feed temperature, with stable performance (16.0 ± 0.3 L/m^2^h) and good distillate quality maintained through simple water flushing. EL-Bourawi et al. [70] experimentally studied ammonia removal via vacuum membrane distillation (*VMD*), examining the effects of the feed flow velocity, temperature, concentration, *pH*, and downstream pressure. They found that *pH* was the dominant factor, achieving >90% removal efficiency with separation factors >8 under optimized conditions. Abdallah et al. [71] experimentally developed a *TNTs* − *PES* blend membrane and tested it in vacuum membrane distillation (*VMD*) for desalination, comparing it to a commercial *PTFE* membrane. They achieved a maximum salt rejection of 98% and flux of 15.2 kg/m^2^h, with the new membrane consistently outperforming *PTFE* in salt rejection (e.g., 99.3% vs. 90.6% at 3000 ppm). Qamar et al. [72] combined a numerical (DNS/CFD) and experimental study. They designed and 3D-printed novel airfoil-shaped (NACA1410) ladder-type feed spacers at two angles of attack (*A* − 10° and *A* − 30°) and compared them with a commercial spacer in a crossflow ultrafiltration setup. They found that the *A* − 30° spacer generated unsteady hydrodynamics/vortex formation, separated wake structures, and recirculation, increased permeate flux by 228%, reduced specific energy consumption by 23%, and reduced biofouling (*OCT*-confirmed) by 74% relative to the commercial spacer. Zhang et al. [73] experimentally developed an IHEVMD module using embedded polymeric PE heat-exchange tubes within a PP membrane bundle for combined in-situ heating and turbulence promotion. The design achieved an 83% flux enhancement, 90% GOR improvement, and 50% STEC reduction over conventional VMD, with strong anti-scaling performance under high-salinity feeds. Kotb et al. [74] experimentally validated a wind-powered multistage VMD system with a water ejector, tested with and without battery storage. It achieved a peak flux of 181 kg/m^2^h and UPC as low as 2.8 $/m^3^ at higher wind speeds. BenAbdallah et al. [75] experimentally investigated a mechanically agitated submerged VMD system using a PVDF hollow fiber module. Agitation increased permeate flux by ~30%, reaching 10.92 kg/h·m^2^ at 70 °C, with HCl-based cleaning proving most effective against fouling.

### 2.5. Aerodynamic Profiles as Passive Mixing Promoters: Research Gap and Present Contribution

The literature reveals an omnipresent constraint: all passive mixing methods published that spacer grids, helical inserts, metal foams, and flow-path re-design achieved boundary-layer mixing by sacrificing hydraulic resistance due to obstruction of the flow. Hence, flux enhancement and pump energy remain coupled, limiting the applicability of passive enhancement in *VMD*. Aerodynamic lifting profiles operate on a different principle: rather than obstruction, an inclined, cambered airfoil generates strong and controllable orifices. This way, the boundary layer can be scraped while maintaining small drag (1 form). Airfoils such as the asymmetric NACA2412 [76] were subjected to *AoA* values of 0°, 5°, 8°, and 12° at *Re* = 1000 and 1500, providing design criteria forenergy-efficient, high-performance *VMD* modules. Prior passive mixers for VMD/MD channels—net-type spacers, helical strakes, metal foams, and even prior airfoil-shaped spacer arrays—function as flow obstructions distributed across the channel, typically incurring pressure-drop penalties in the hundreds of Pa to kPa range. In contrast, the present work isolates a single, non-obstructing cambered NACA 2412 element and systematically varies its angle of attack and orientation, achieving a 14.6% flux gain at a pressure-drop penalty of only ≈10 Pa. To our knowledge, this is the first systematic angle-of-attack/orientation study of a discrete cambered airfoil, rather than an airfoil-shaped spacer array, as a VMD mixing promoter.

## 3. Numerical Methodology

The CFD simulation of the hydrodynamic and heat-transfer performance of the NACA 2412 airfoil inserted into the *VMD* feed channel has been performed using a two-dimensional *k* − *ω* steady-state model in Ansys Fluent 2025 R2 (Ansys, Inc., Canonsburg, PA, USA). The geometric setup of the domain, solving equations, boundary settings, meshing approaches, and solver settings are detailed below.

### 3.1. Geometric Domain and Mixing Promoter Configuration

The simulation domain is a typical flat-sheet *VMD* channel flow setup. The channel length is *L* = 60 mm, and the height *H* = 3 mm. A passively implemented vortex generator in the form of a cambered NACA 2412 airfoil with a *c* = 5 mm chord length lies inside the channel. To analyze spatial mixing in an organized manner, the angle of the airfoil, *AoA* (α), is varied upward and downward for cases = 5°, 8°, and 12°, yielding seven configurations plus *AoA* = 0°. The NACA 2412 profile was selected as a moderately cambered, well-characterized asymmetric section with extensive documented aerodynamic reference data [76], providing a traceable benchmark geometry for this first systematic exploration of camber- and incidence-driven passive mixing in VMD channels. The AoA values (5°, 8°, and 12°) span a graded incidence range intended to reveal trends in the flux enhancement and hydraulic penalty with an increasing angle of attack; this study is a parametric/exploratory investigation establishing feasibility and design trends for this mixing strategy rather than a formal shape- or angle-optimization study, with systematic optimization identified as future work (Section 5). The Reynolds numbers investigated (Re = 1000 and 1500) were selected to fall within the representative operating range reported for flat-sheet VMD/MD channels in the literature (e.g., Re = 400–1500 in [47]) and to remain consistent with the range over which the present numerical model is validated against independent data (Re = 200–1000, Section 4.2). The simulation domain is shown schematically in Figure 1.

### 3.2. Governing Equations

The feed fluid (saline water) is treated as an incompressible, non-Newtonian fluid with temperature-dependent physical properties. Steady-state transport is governed by the following conservation equations, expressed in vector form:

**Continuity:**

∇ · (*ρ u*) = 0 (1)

**Momentum:**

∇ · (*ρ uu*) = −∇*p* + ∇ · [*μ* (∇*u* + (∇*u*)*ᵀ*)] (2)

**Energy:**

∇ · (*ρ c_p_ u T*) = ∇ · (*k* ∇*T*) (3)

**Mass transport:**

∇ · (*u C*) = ∇ · (*D* ∇*C*) (4)

where *u* is the velocity vector, *ρ* is the fluid density, *p* is pressure, *μ* is dynamic viscosity, *c*_p_ is the specific heat capacity, *k* is thermal conductivity, *C* is the local solute concentration, and *D* is the mass diffusion coefficient of salt in water.

### 3.3. Boundary Conditions and Membrane Interface Modeling

The boundary conditions, summarized in Table 1, replicate a realistic thermal desalination configuration. The feed enters at a uniform velocity set by the target Reynolds number, at 80 °C and with seawater salinity (35,000 ppm), and leaves through a zero-gauge-pressure outlet. The bottom wall and the airfoil surfaces are treated as no-slip, adiabatic, and impermeable, so heat and mass leave the channel only through the membrane interface at the top wall.

The membrane interface acts as a simultaneous heat and mass sink. The trans-membrane vapor flux is computed from the vapor-pressure difference across the membrane:

*J* = *K_m_* · (*P_m_*_,*f*_ − *P_m_*_,*p*_) (5)

where *K_m_* is the membrane permeability coefficient, *P_m_*_,*f*_ is the partial vapor pressure at the feed–membrane interface evaluated from the Antoine equation at the local interface temperature *T_m_* and water salinity *C*, and *P_m_*_,*p*_ is the constant permeate-side vacuum pressure (*P_v_* = 5 kPa). The corresponding latent heat *q_m_* = *J* · Δ*H*_vap_ is removed at the interface, and complete solute rejection is enforced (*J_s_* = 0). Hence, the salt rejected by the membrane accumulates in the concentration boundary layer, thereby driving the *CPC* reported in the results.

The mean free path of water vapor greatly exceeds the membrane pore radius, so molecule–wall collisions dominate the pore transport, and the membrane permeability is evaluated with the Knudsen-viscous diffusion model:

(6) $$K_{m}\;=\;\left(\frac{2\pi }{3}\right)\left(\frac{1}{RT}\right){\left(\frac{8RT}{\pi M_{w}}\right)}^{\frac{1}{2}}(r^{3}/\delta \tau )$$

where *r*, *δ*, and *τ* are the membrane pore radius, thickness, and tortuosity, *M_w_* is the molecular weight of water vapor, and *R* is the universal gas constant. The partial vapor pressure of the saline feed at the membrane interface is obtained from an Antoine expression corrected for the presence of dissolved salt [7]:

*P_m_*_,*f*_ = *exp*(23.1964 − 3816.44/(*T* − 46.13)) / [1 + 0.57357 (*C*/(1000 − *C*))] (7)

where *T* (K) and *C* (g/kg) are the local temperature and solute concentration at the feed–membrane interface. Finally, the temperature and concentration polarization coefficients used throughout the results are defined as follows:

(8) $$TPC\;=\;\frac{T_{m,f}}{T_{f}}$$

(9) $$CPC=\frac{C_{m,f}}{C_{f}}$$

where *T_m_*_,*f*_ and *C_m_*_,*f*_ are the temperature and solute concentration at the feed-side membrane surface, and *T_f_* and *C_f_* are the corresponding feed inlet values. The *TPC* is bounded above by unity, with lower values indicating stronger thermal polarization and a larger loss of evaporative driving force. In contrast, the *CPC* is bounded below by unity, with higher values indicating stronger solute accumulation at the interface.

### 3.4. Mesh Generation and Grid Independence Study

An excellent-quality, fully unstructured mesh was created to ensure grid independence and capture adequate steepness of velocity, temperature, and concentration gradients in the boundary layer. Inflation layers were introduced around the membrane and all airfoil boundaries, with the mesh wall spacing set to ensure *y*^+^ < 1 under all tested operating conditions (Figure 2).

Grid dependence of selected mesh densities are reported (*M*1 = 35,000, *M*2 = 70,000, *M*3 = 140,000, *M*4 = 240,000 elements). The trans-membrane flux (*J*), *TPC*, and *CPC* have been considered measures of grid convergence, and values are presented in Table 2, together with graphs illustrating differences (Figure 3). Between the *M*3 and *M*4 meshes, flux differences of less than 0.5% are observed, suggesting minimal benefit from further refinement to that level. Therefore, the *M*3 mesh (140,000 elements) has been used as the optimal mesh configuration to balance acceptable computational cost and the resolution of localized flow structures, such as flow separation and wake recirculation around the airfoil.

### 3.5. Numerical Schemes and Convergence

The governing equations were discretized and computed with the Ansys Fluent pressure-based solver using the Coupled pressure–velocity algorithm to achieve stable, robust convergence. The second order upwind discretization scheme was utilized for the momentum, energy, and mass transport equations to have higher-order accuracy spatially for minimum numerical diffusion, and the residual equations for residuals less than 10^−3^ were considered converged and 10^−6^ for the energy equation.

## 4. Results and Discussion

### 4.1. Grid-Independence Validation

A sample of the grid-independence results is presented in Table 2 and Figure 3. As can be observed, the values *y*^+^ on both sides of the airfoil (*y*^+^ ≤ 0.205) and on the membrane (*y*^+^≤ 0.119) all show adequate resolution of the viscous sublayer, even in the fine-mesh configurations. The relatively low 0.22% variability in flux between the chosen fine-mesh and ultra-fine-mesh *M*3/*M*4 justified selecting mesh *M*3 (140,000 elements) for all subsequent parametric analyses. The remainder of this paper uses data produced using mesh density *M*3. To quantify the numerical uncertainty associated with mesh discretization, the Grid Convergence Index (GCI) was computed following the procedure of Celik et al. [77] using the flux values from meshes M2–M4 (Table 2). The apparent order of convergence was found to be *p* ≈ 0.89, yielding a Richardson-extrapolated flux of 56.99 kg/m^2^h and a discretization uncertainty of GCI ≈ 1.05% on the production mesh (M3). This uncertainty is applied as a ±1.05% band on all flux values reported in Tables 5 and 6. All reported AoA- and orientation-driven flux changes exceed this band by at least an order of magnitude (the smallest effect, +14.6% at upward AoA = 12°, Re = 1500, is ~14× the numerical uncertainty), confirming that the observed trends are numerically well-resolved rather than discretization artifacts.

### 4.2. Model Validation

The current CFD model is validated against the experimental and numerical results previously obtained by Anqi et al. [78]. For validation, we considered the ideal conditions of a steady-state, incompressible flow in a single flat-sheet *VMD* channel with no spacer filaments, and we selected the boundary conditions as follows. A comparison of average trans-membrane flux from our model with values reported by Anqi et al. [78] is shown in Table 3 across the range of test conditions. These conditions vary with Reynolds numbers from 200 to 1000 and with feed temperatures of 50 °C or 80 °C. We observe good agreement between the two sets of results: a minimum percent difference of 2.20% for *Re* = 1000, *T* = 50 °C and a maximum percent difference of 18.05% for *Re* = 200, *T* = 80 °C. We get within 5.5% in 3 out of 5 cases. The model, thus calibrated, will be used to conduct numerical investigations of the effects of various operating parameters in the section below.

This validation confirms the fidelity of the solver, mesh, and turbulence-model settings for predicting VMD permeate flux under empty-channel conditions. As the airfoil-in-channel configuration investigated in this study is a novel design not yet realized experimentally, direct experimental validation of the airfoil-induced flow patterns, pressure drop, and flux enhancement reported in Section 4.4 is not yet available; the present results should accordingly be interpreted as CFD-based predictions pending future experimental confirmation (see Section 5).

### 4.3. Comparative Performance Analysis

The parametric analysis included varying the NACA 2412 airfoil *AoA* in upward and downward orientations at Reynolds numbers of *Re* = 1000 and 1500, respectively, as the *AoA* = 0° (airfoil without rotation). For each Reynolds number, we establish the zero-incidence case as the reference. Here, we present the permeate flux *J*, the temperature polarization coefficient (*TPC*), the concentration polarization coefficient (*CPC*), and the channel pressure drop ∆*P* in Table 4, Table 5 and Table 6, respectively. The configuration was chosen based on the criterion that the system should achieve the best performance in *J*, *TPC*, *CPC*, and ∆*P*.

The channel pressure drop reflects two additive contributions: viscous wall friction, which scales linearly with bulk velocity, and the form (profile) drag of the airfoil itself, which scales approximately with the square of velocity. At Re = 1000, the CFD-predicted baseline pressure drop (2.72 Pa) closely matches the analytical Hagen–Poiseuille prediction for friction-dominated flow between parallel plates (≈1.7 Pa), with the small excess attributable to the airfoil’s blockage and entrance effects. At Re = 1500, the baseline pressure drop (13.33 Pa) exceeds the friction-only estimate by a larger margin, consistent with a growing form-drag contribution as velocity increases. In all cases, the absolute pressure drop remains 2–3 orders of magnitude below the several-hundred-Pa-to-kPa range typical of conventional VMD spacers and vortex generators [45,57], confirming that the proposed design imposes a comparatively minor hydraulic penalty even after accounting for airfoil form drag.

To more rigorously evaluate the flux enhancement reported above against its associated hydraulic penalty, we define a Merit Number, $Me=\left(\frac{J}{J_{0}}\right)\cdot {\left(\frac{\Delta P_{0}}{\Delta P}\right)}^{\frac{1}{3}}$, where *J*_0_ and Δ*P*_0_ denote the zero-incidence baseline flux and pressure drop at the corresponding Reynolds number. This formulation follows the standard Performance Evaluation Criterion (PEC) approach used to compare transport enhancement against a pumping-power penalty under a constant-pumping-power constraint. Table 7 reports Me for upward and downward configurations at *Re* = 1000 and 1500, for *AoA* = +12 and −12.

All configurations yield Me < 1, indicating that, in strict proportional terms, the pressure-drop penalty outpaces the flux gain. This result is a direct consequence of the very low baseline pressure drop in this laminar VMD channel (2.72–13.33 Pa); any absolute pressure increase, however small, registers as a large relative penalty against such a small reference value, a known limitation of ratio-based merit indices when applied near a zero baseline. Considered in absolute terms, the added pressure drop remains modest (≤23.5 Pa across all tested configurations) relative to the kilopascal-scale transmembrane vacuum pressures typical of VMD operation, so the corresponding increase in pumping energy demand is minor in absolute magnitude even where it is proportionally large. We report Me alongside the absolute values in Table 5 and Table 6 so that flux–pressure-drop trade-offs can be assessed from both a normalized and an absolute perspective.

Figure 4, Figure 5, Figure 6 and Figure 7 present local profiles of permeating flux, *TPC*, and *CPC* along the membrane surface, corresponding to the aggregate results in Table 5 and Table 6, respectively, with the baseline 0° incidence profile included for reference in each case. In all cases, the profiles are similar in shape. The permeate flux is highest near the start of the channel, where the thermal boundary layer is still thin and offers minimal resistance to vapor transport, and it decreases along the membrane as the boundary layer builds up toward the outlet. The *TPC* follows this same trend: it is near its ideal value of 1 at the inlet, where the thin boundary layer keeps the membrane-surface temperature close to the bulk feed temperature, and it decreases along the membrane as the growing boundary layer increases the temperature drop across it. The *CPC*, in contrast, moves in the opposite direction, starting near 1 at the inlet before any salt has accumulated on the membrane surface, and increasing along the membrane toward the outlet as rejected salt accumulates with continued permeation. The absence of a response in *CPC* to the angle of attack across all four diagrams is further evidence that concentration polarization is negligible and plays a far smaller role than temperature polarization, so the flux increase is driven entirely by *TPC* changes. We can therefore see how the NACA 2412 airfoil can enhance permeate flux and *TPC*, with *CPC* remaining largely unaffected. Because the airfoil’s benefit operates through renewal of the thermal boundary layer rather than disruption of the salt-concentration layer at the membrane surface, the reported flux enhancement is expected to persist for high-salinity feeds. However, the airfoil does not by itself mitigate concentration-polarization-driven scaling, and feeds with high scaling propensity would still require complementary anti-scaling measures alongside this geometry; experimental validation will be needed to determine the extent to which these CFD-based conclusions hold.

Figure 4 shows the upward orientation at *Re* = 1500. At the airfoil outlet, downstream of the boundary layer, the local permeate flux profile diverges as the incidence increases: at incidences higher than the baseline, the permeate flux (a) and consequently the *TPC* (b) increase continuously along the membrane compared with the baseline profile, with the average flux increasing from 57.60 to 66.03 kg/m^2^h, the *TPC* increasing from 0.9319 to 0.9381, and the CPC varying from 1.0017 to 1.0019, all at an incidence of 12°. The physical explanation is that flow entering the suction surface of the airfoil deflects toward the membrane, maintaining higher energy and higher temperatures in contact with the membrane and preventing the formation of a thick boundary layer over the back half of the channel, where the airfoil’s influence would otherwise be lost. This lift effect on the permeate flux near the end of the channel is responsible for the 14.6% permeate flux enhancement reported in Table 5.

Figure 5 repeats the comparison at *Re* = 1000. The profiles have the same shape, but the enhancement is weaker: at 12° the flux rises from 51.74 to 57.27 kg/m^2^h (+10.7%, versus +14.6% at *Re* = 1500) and the TPC from 0.9275 to 0.9318, with the CPC increasing from 1.0021 to 1.00234. With less momentum in the incoming stream, the airfoil has less kinetic energy to redirect toward the membrane, so the boundary-layer renewal that it provides is milder, a direct illustration of why the airfoil and a higher feed Reynolds number are coupled rather than independent.

Figure 6 shows the downward orientation at *Re* = 1500. Unlike the upward case, the gain at −12° is much smaller than its upward counterpart: average flux rises from 57.60 to only 62.20 kg/m^2^h (an 8.0% increase, versus 14.6% for upward at +12°) and TPC from 0.9319 to 0.9350, despite paying a comparable pressure drop of 23.38 Pa (versus 23.52 Pa for upward at +12°). At the smaller angles, the picture is worse still: flux at −5° and −8° falls slightly below the baseline, to 56.80 and 56.45 kg/m^2^h, respectively, with *TPC* also falling slightly below the baseline. This underperformance conceals a more complex local picture. Because the pressure surface faces the membrane, the fluid first enters a convergent channel between the airfoil and the membrane, briefly boosting local flux above the baseline. This gain is short-lived: by roughly the channel midpoint, the flow separates into a deficient wake region where local flux and *TPC* drop well below their baseline values, before the flow reattaches and recovers somewhat near the outlet. Fewer local variations are seen at the smaller angles, where the −5° and −8° profiles fall slightly below or track the baseline over almost the entire channel length. In short, the strong local peaks seen at −12° are largely offset by the deficit in the wake region, so the net gain at that angle is modest despite real local enhancement over part of the channel, while at the smaller angles, local losses outweigh local gains entirely.

Figure 7 illustrates a deeper, more negative wake penalty at *Re* = 1000 than at *Re* = 1500. The post-airfoil pressure dip for the −12° case now lies well below the baseline minimum, indicating that this is the most deeply depressed low-pressure region seen across the study. At −5° and −8°, average flux (50.40 and 49.43 kg/m^2^h, respectively) falls below the baseline case (51.74 kg/m^2^h) a larger relative deficit than the equivalent angles saw at *Re* = 1500, despite the −12° case still paying the pressure penalty associated with going deep into this negative-pressure trough. At lower velocities, the wake region behind the airfoil becomes larger and persists further downstream before dissipating, so more momentum is lost before the flow can recover near the outlet. Combined with Figure 6, Figure 7 shows why orientation choice matters so much at lower Reynolds numbers: without enough incoming momentum, the downward configuration cannot reliably recover from the wake it creates, whereas the upward orientation avoids this penalty entirely by keeping the accelerated flow on the membrane side of the airfoil.

Figure 8 delineates the local velocity field along the Y-axis for airfoil 3 across distinct flow regimes characterized by Reynolds numbers of 1000 and 1500 and angles of attack of 0°, +12°, and −12°. Subfigure (a) captures the pre-airfoil local velocity distribution at *x* = 0.02 mm, illustrating the upstream flow conditions and the immediate boundary layer behavior prior to surface interaction. Conversely, subfigure (b) evaluates the post-airfoil flow field at *x* = 0.025 mm, where significant velocity deficits, wake development, and flow asymmetry become apparent as a function of the varying aerodynamic loading. The pronounced divergence between the upstream and downstream profiles highlights the sensitivity of local momentum dispersion, flow separation, and wake dynamics to changes in both the angle of attack and Reynolds number scaling.

### 4.4. Hydrodynamic Mechanisms and Discussion

The upward orientation consistently beat both the baseline and the downward orientations at all test Reynolds numbers. The CFD simulations used a steady-state k-ω turbulence model after verifying mesh independence. With a cambered NACA 2412 profile serving as an effective flow guide, the interaction with the geometry in the upward arrangement creates a nozzle effect between the airflow on the airfoil’s suction surface and the membrane, thereby accelerating flow onto the membrane surface and producing coherent separated wake structures that enhance near-wall flow renewal and disturb the thermal boundary layer. This is illustrated by the progressive increase in *TPC* from 0.9319 at baseline to 0.9381 at 12°, resulting in a 14.6% increase in flux (from 57.60 to 66.03 kg/m^2^h at *Re* = 1500), highlighting the effectiveness of the aerodynamic method. There is a hydraulic cost associated with this improvement: for increasing the incidence from 5° to 12°, the pressure drop steadily increased from 14.73 to 23.52 Pa only on the order of 10 Pa in absolute terms—and the flux enhancement per unit pressure increase was significantly more favorable than for flow-obstructive elements, since the flow deflection produced by the streamlined profile kept the drag increase relatively small even at these higher incidence angles.

In contrast, the downward orientation yields less benefit for the same hydraulic input: because the pressure side faces the membrane, the flow is deflected away, and near-wall convection decreases. The pressure drop is quite comparable at *Re* = 1500, and pressures at 5°, 8°, and 12° (14.46, 16.86, and 23.38 Pa) fall within about 3% of the equivalent upward values, but flux is consistently lower, and less consistently so: at −5° and −8°, flux (56.80 and 56.45 kg/m^2^h) falls slightly below the baseline of 57.60; even at −12° (62.20 kg/m^2^h), flux remains below its upward equivalent (66.03 kg/m^2^h). The same pattern repeats at *Re* = 1000, where the −5° and −8° cases (50.40 and 49.43 kg/m^2^h) again fall below the baseline of 51.74 kg/m^2^h. The downward orientation therefore accepts the pressure cost of the inclined profile without capturing the mixing advantage at the membrane, confirming that the directional flow, not the mere presence of blockage, is what generates flux from hydraulic input. This finding aligns with Alwatban et al. [47], who concluded that the temperature polarization coefficient (*TPC*) is the major limitation on permeate flux; this study demonstrates a robust, geometry-driven approach to mitigating that limitation. Unlike the metal foam inserts used by Loussif and Orfi [45] to promote hydraulic mixing by disrupting boundary layers typically at a high pressure penalty, the current airfoil strategy offers a significantly better trade-off: the maximum pressure drop achieved here (23.52 Pa, at *Re* = 1500 and 12°) is roughly two orders of magnitude smaller than the 3332.5 Pa (3.3325 kPa) reported using metal foam [45] at a comparable operating condition, while still achieving a double-digit percentage flux gain. Limiting this hydraulic mixing cost to such a small figure represents an important step toward efficient membrane channel design.

In a comparison with Qamar et al. [72], using airfoiled NACA1410-type ladders as crossflow spacers in ultrafiltration, our A–30 spacer caused vortex formation, separated wake structures, and recirculation to destabilize around AoA; it increased the permeate flux and reduced specific energy consumption for water transport by 228% and 23%, respectively, when compared to a commercial spacer. This resulted mainly from decreased biofouling levels (74% reduction in OCT-measured bacterial cells), but not purely from a hydrodynamic factor. The milder flux gain found in our work of 14.6% at Re = 1500 with this specific spacer configuration is driven by a totally different transport-limiting mechanism, since we are working with a heat-driven VMD process. For this work, the mechanism relies upon a reduction of temperature polarization and not biofouling. Even though they concluded that their system was controlled by the concentration difference for mass transfer (biofouling is one dominant cause of this), the difference between two studies highlights both that any form of an aerodynamic AoA controlled surface flow pattern, which modifies the fluid circulation near the boundaries, may work in both UF and VMD-type membrane separations with both kinds of spacers, provided that this aspect modifies the relevant controlling mass/heat transport mechanism (though, this implies different pressure loss contributions). Our study indicates a large potential at low pilot-scale pressure drops, compared to similar mechanisms reported for UF, where the fluid pressure drops must be increased and controlled for full-scale development in UF. While the present study does not simulate fouling directly, the mechanism responsible for the observed flux enhancement increased near-wall shear and reduced boundary-layer residence time parallels that were identified by Qamar et al. [72] as responsible for a 74% reduction in biofouling attachment in an airfoil–spacer configuration, suggesting the present design may offer a comparable anti-fouling benefit, pending experimental confirmation.

Temperature and velocity profiles show local behaviors in Figure 9, Figure 10, Figure 11 and Figure 12. These local behaviors underlying distributions for temperature and velocity are shown in terms of contour plots, where each compartment compares the zero-incidence (reference) profile with all three upward and three downward cases. In the contours, temperature displays a hot feed and a cold film along with the membrane because the evaporation process removes latent heat from the membrane. This temperature polarization at the membrane interface means that the cold film represents heat removal across the membrane, and its width represents the driving force removed.

Figure 9 shows the temperature distribution at *Re* = 1000. In the reference (baseline) setup, this cold film gently undulates over the airfoil surfaces and progressively thickens downstream along the membrane length. In the upward configuration, the hot feed flow is pressed toward the membrane, producing a thin, uniform film along the array; at 12°, the film is so thin that the bulk temperature is maintained at the membrane surface for almost the entire length of the channel, consistent with the TPC enhancement reported in Table 6. In the downward configuration, the reverse occurs: stagnant, cooled fluid collects behind each airfoil, separated from the stream that has already spent its heat at the membrane upstream, producing a lumpier, thicker film layer.

Figure 10 illustrates the same phenomenon as Figure 8, but at the higher Reynolds number of *Re* = 1500, where the effect is more pronounced. As incidence increases, the cooling film thins further, and at 12°, it becomes virtually absent over most of the array—flux stays suppressed until just past the last airfoil, where it begins to recover, consistent with the flux enhancement reported in Table 5 for the 12° case. At the smaller angles, however, the cold wakes behind each airfoil remain deeper and more sharply defined, corresponding to the local TPC minimum seen in the downward orientation profiles of Figure 6: the stagnant fluid trapped at the membrane surface behind each blade no longer contributes meaningfully to evaporation.

Figure 11 shows the velocity field at Re = 1000. In the baseline configuration, the flow is symmetric: fluid accelerates above and below each airfoil (shown in the shaded regions), leaves a small low-speed region behind each airfoil tip, then relaxes back toward the upstream velocity without preferentially accelerating toward either the membrane or the bottom wall. When the airfoils are rotated, this symmetry breaks down. At a positive incidence, the band of accelerated flow along the membrane is enhanced and passes from one airfoil to the next, while low-speed separated flow sheds toward the pressure surface of each airfoil. At negative incidence, the accelerated band instead appears along the bottom wall, with flow recirculating in low-speed patches directly beneath the membrane. This membrane-band effect is less intense at this Reynolds number than at the higher-Re case.

Figure 12 shows the velocity field at *Re* = 1500, where the directional asymmetry is at its strongest. At 12°, the flow consists of a wall-jet traversing the full array at high speed; the nozzle effect invoked earlier becomes a physical reality accompanied by small separation zones on the pressure surface of each airfoil. At 12°, the flow is the mirror image: the wall-jet forms along the bottom wall instead, with stagnation regions adjacent to it. This stagnation region corresponds to the local flux minimum reported in Figure 6. Taken together, these two cases reinforce the paper’s central finding: upward and downward orientations experience nearly the same pressure loss, but only the upward orientation produces a wall-jet on the membrane surface.

Figure 13 presents a close-up streamline view around the airfoil elements for the two extreme configurations, upward orientation at *AoA* = 12° and downward orientation at *AoA* = −12°, both at *Re* = 1500 and *T_in_* = 80 °C. In the upward case, the streamlines confirm the wall-jet mechanism proposed earlier: flow accelerates continuously along the membrane (top wall), reaching its highest velocities (up to ≈0.21 m/s) directly above each airfoil’s suction surface, with the jet re-forming and persisting from one airfoil to the next along the full length of the channel. A small low-velocity recirculation region is visible on the pressure side of each airfoil, but it remains confined to the lower half of the channel, away from the membrane, so it does not interrupt the accelerated flow feeding the mass-transfer surface. In the downward case, the streamline pattern mirrors this behavior about the channel centerline: the high-velocity band instead forms along the bottom (non-membrane) wall, while the flow on the membrane side of each airfoil’s suction surface slows and locally recirculates. This membrane-side stagnation is most pronounced immediately downstream of the leading airfoil, consistent with the local flux minimum identified in Figure 6, and explains why the downward orientation, despite incurring a nearly identical pressure-drop penalty to the upward case, yields a smaller net flux enhancement: the same aerodynamic deflection that intensifies mixing at the membrane in the upward orientation instead directs the accelerated flow away from it when the airfoil is rotated in the opposite sense.

## 5. Conclusions

Herein, we conducted a comprehensive numerical CFD investigation of the hydrodynamic and mass-transfer performance of an NACA 2412 airfoil used as a passive mixing element in the feed channel of a VMD system. Through a parametric analysis of the angle of attack and Reynolds number, we established a pronounced directional asymmetry in performance with respect to the airfoil orientation. The upward configuration (positive incidence) directs high-momentum flow toward the membrane surface, disrupting the thermal and concentration boundary layers and enhancing mass transfer: at *Re* = 1500, TPC increased from 0.9319 at baseline to 0.9381 at 12°, while permeate flux rose from 57.60 to 66.03 kg/m^2^h a 14.6% increase with channel pressure drop rising from 13.33 to 23.52 Pa. At *Re* = 1000, the same trend held at a reduced magnitude: flux rose from 51.74 to 57.27 kg/m^2^h (a 10.7% increase). This maximum pressure drop remains roughly two orders of magnitude smaller than the pressure penalty reported for metal-foam insertion methods [45] under comparable conditions.

The upward configuration at 12° achieved the maximum permeate flux at both tested Reynolds numbers (66.03 kg/m^2^h at *Re* = 1500 and 57.27 kg/m^2^h at *Re* = 1000) and consistently generated more flux per unit pressure drop than the downward configuration, making it the more reliable enhancement strategy across the parameter range tested. Although the downward orientation experiences a nearly identical pressure-drop penalty to the upward orientation at matching incidence magnitudes, it produces a smaller flux gain overall, and at low incidence angles (−5°, −8°), flux falls slightly below the zero-incidence baseline. This confirms that the direction of flow deflection relative to the membrane, rather than the presence of the airfoil alone, is the key factor governing performance.

Overall, these results indicate that a cambered NACA 2412 airfoil oriented in the ‘upward’ direction provides significant boundary-layer disturbance with a relatively low pressure-drop penalty and thus bypasses the energy requirement and permeate generation trade-off that previously made passive mixing techniques less attractive for VMD designs. The investigation should be continued with a 3D CFD analysis of a module including several airfoils to verify the results, and the long-term behavior under fouled membrane conditions should be analyzed. Experimental validation of the airfoil-in-channel configuration including a direct measurement of local flow patterns (e.g., via PIV), the pressure drop, and permeate flux is identified as the essential next step to confirm the CFD-based trends reported here before this design can be considered for practical VMD module implementation. Extending the present analysis to a broader Reynolds-number range, spanning lower flow rates, as well as transitional and turbulent regimes beyond Re = 1500, is likewise identified as a priority for establishing the general applicability of the proposed design across realistic VMD operating conditions. The present study is limited to a steady-state, clean-membrane analysis and does not evaluate long-term fouling, scaling, or membrane-wetting behavior, which are critical for practical VMD deployment; while the airfoil’s boundary-layer-disrupting mechanism is consistent with the anti-fouling behavior reported for related passive-mixing designs [72], an experimental assessment of fouling, scaling, and wetting under sustained operation is identified as an essential direction for future work. While the airfoil’s boundary-layer-disrupting mechanism is consistent with anti-fouling behavior reported for related passive-mixing designs [72], an experimental assessment of fouling, scaling, and wetting under sustained operation is identified as an essential direction for future work. Fabrication and module-scale integration of the proposed design also warrant dedicated study: while discrete airfoil-shaped elements at a comparable scale have been fabricated via micro-CNC machining or 3D printing in related spacer designs [68,72], scaling the present single-element configuration to a full VMD module raises additional questions of the array density, spanwise uniformity, and mechanical mounting that fall outside the scope of the present 2D, single-element analysis and should be addressed in the planned 3D, module-scale investigation. The reported gains assume an idealized two-dimensional airfoil profile with a precisely controlled angle of attack. In practical fabrication, tolerances in the blade profile and mounting angle, together with three-dimensional end effects and proximity to the channel side walls, could modestly attenuate the nozzle effect responsible for the upward configuration’s performance. These manufacturing and installation sensitivities should be quantified experimentally before scale-up.

## Figures and Tables

**Figure 1:**
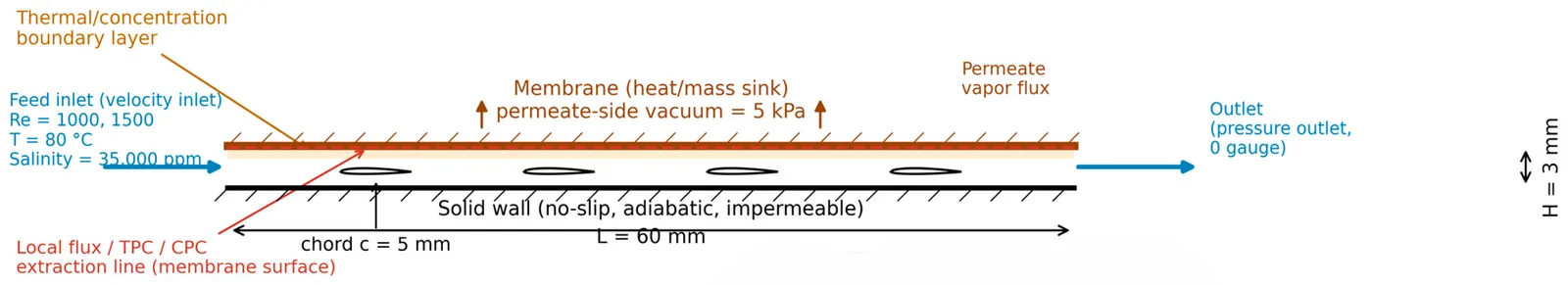
Computational domain geometry: zero-incidence airfoil (*AoA* = 0°, baseline); upward orientation (positive *AoA*); downward orientation (negative *AoA*). *H* = 3 mm, *L* = 60 mm, *c* = 5 mm.

**Figure 2:**
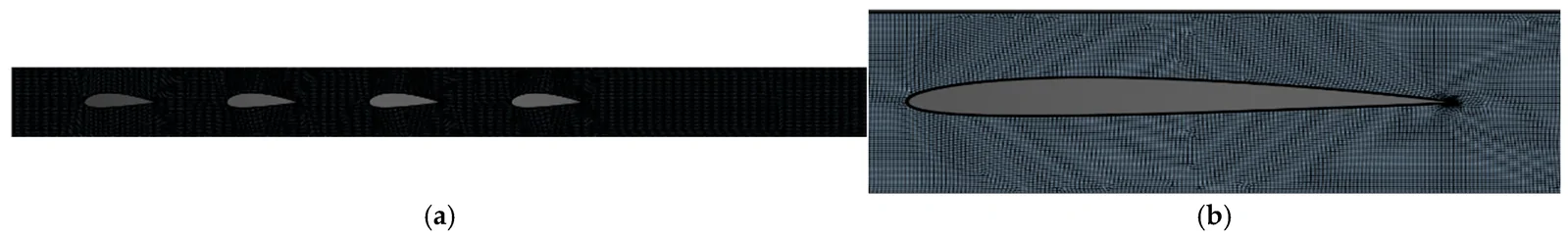
Structured quadrilateral mesh: (**a**) full channel view showing airfoil positioning; (**b**) inflation layers around the NACA 2412 airfoil surface and near-wall refinement at the membrane interface.

**Figure 3:**
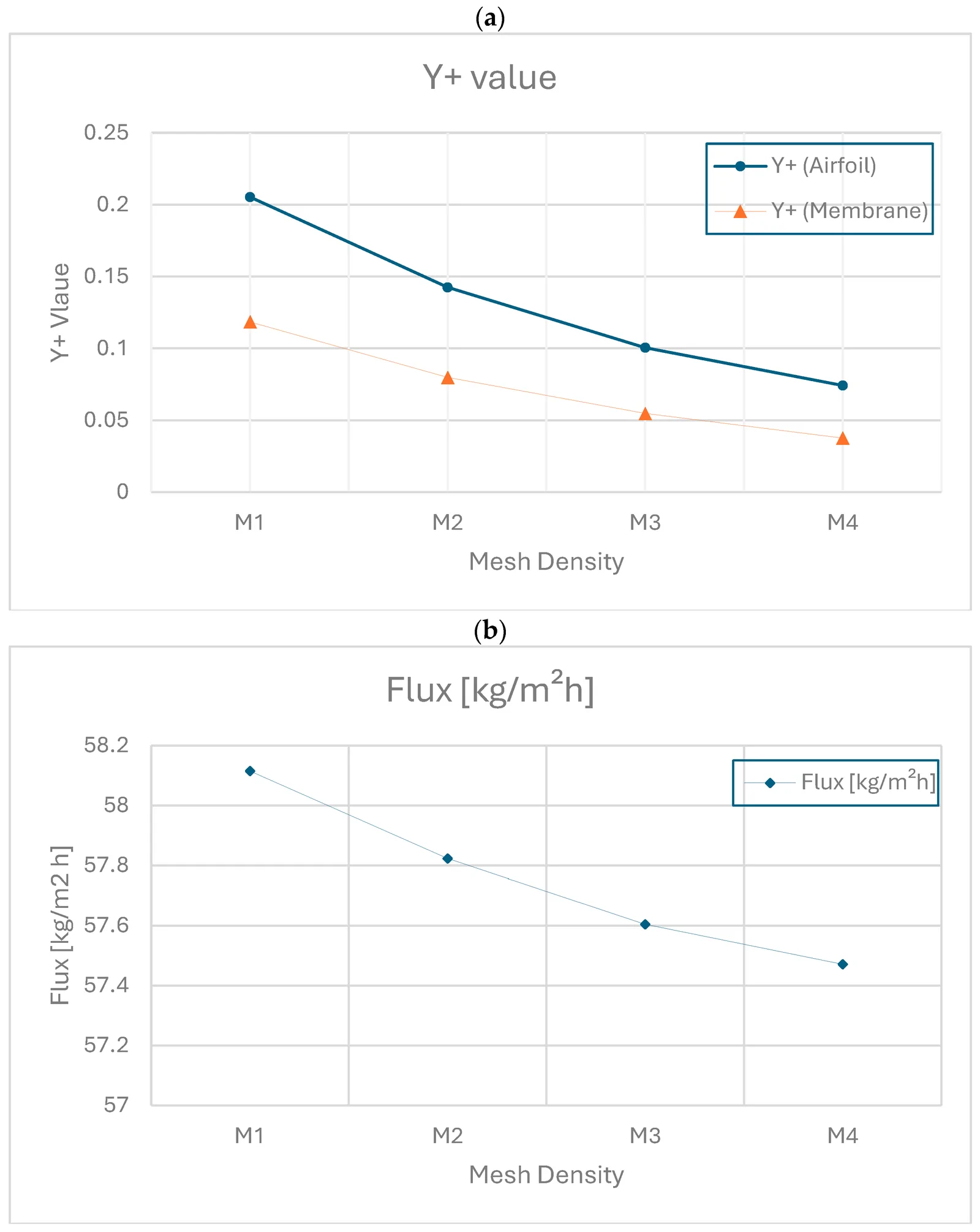
Grid independence results: (**a**) trans-membrane flux and (**b**) *y*^+^ values at the airfoil and membrane surfaces as a function of the mesh element count.

**Figure 4:**
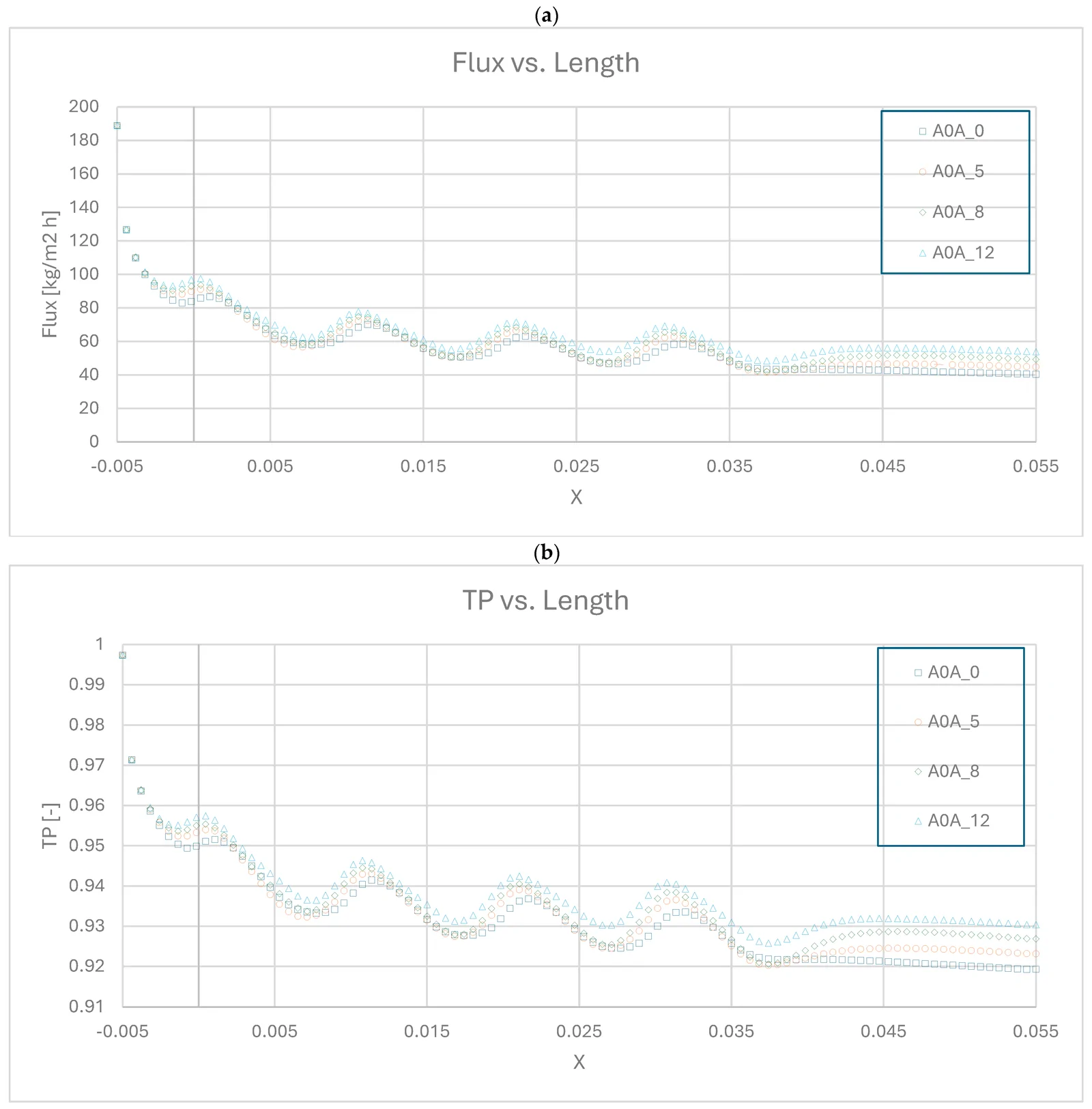

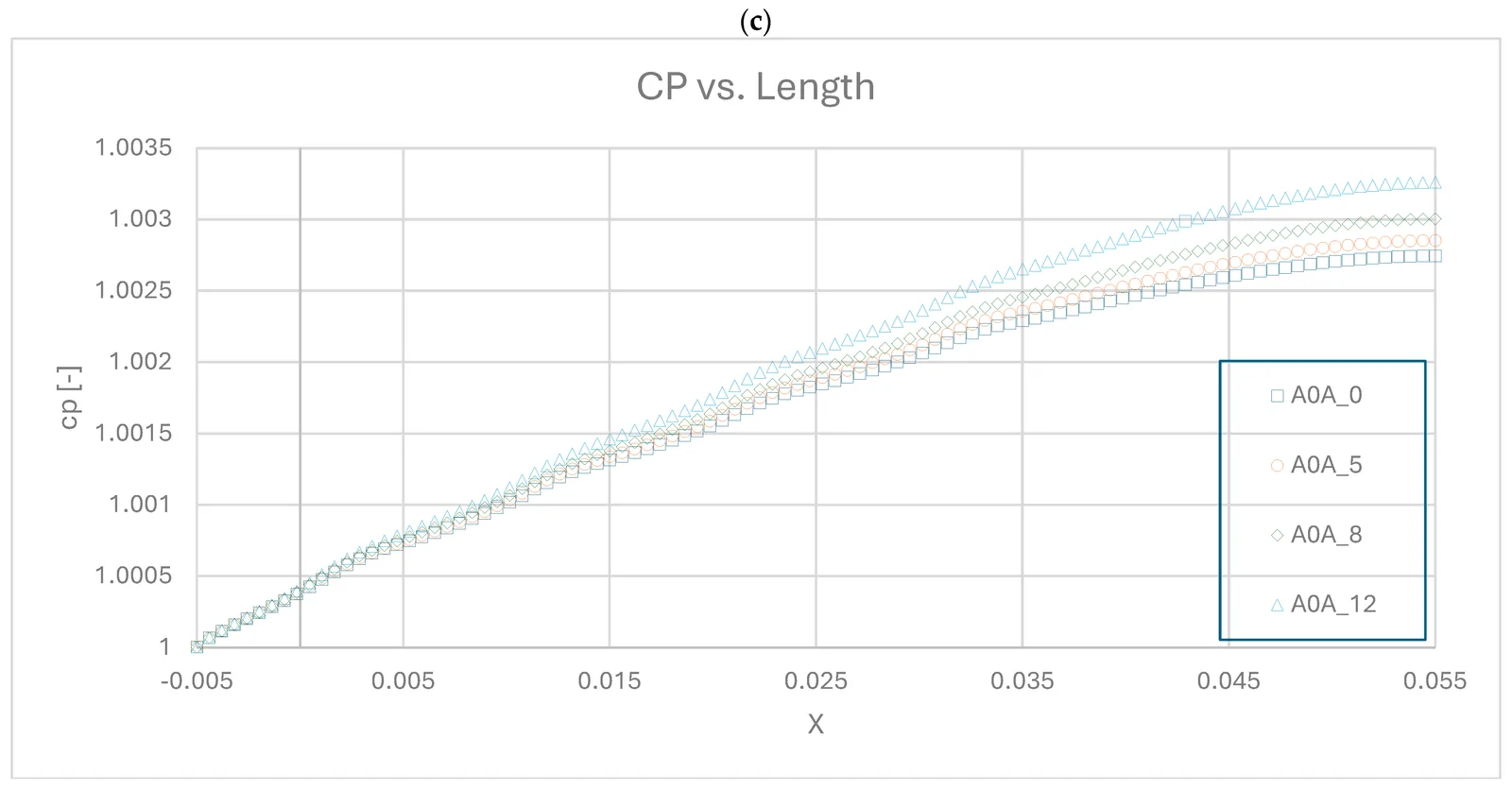
Local profiles of (**a**) permeate flux, (**b**) TP, and (**c**) CP along the membrane surface for the upward orientation at Re = 1500 (AoA = 0° baseline, 5°, 8°, and 12°).

**Figure 5:**
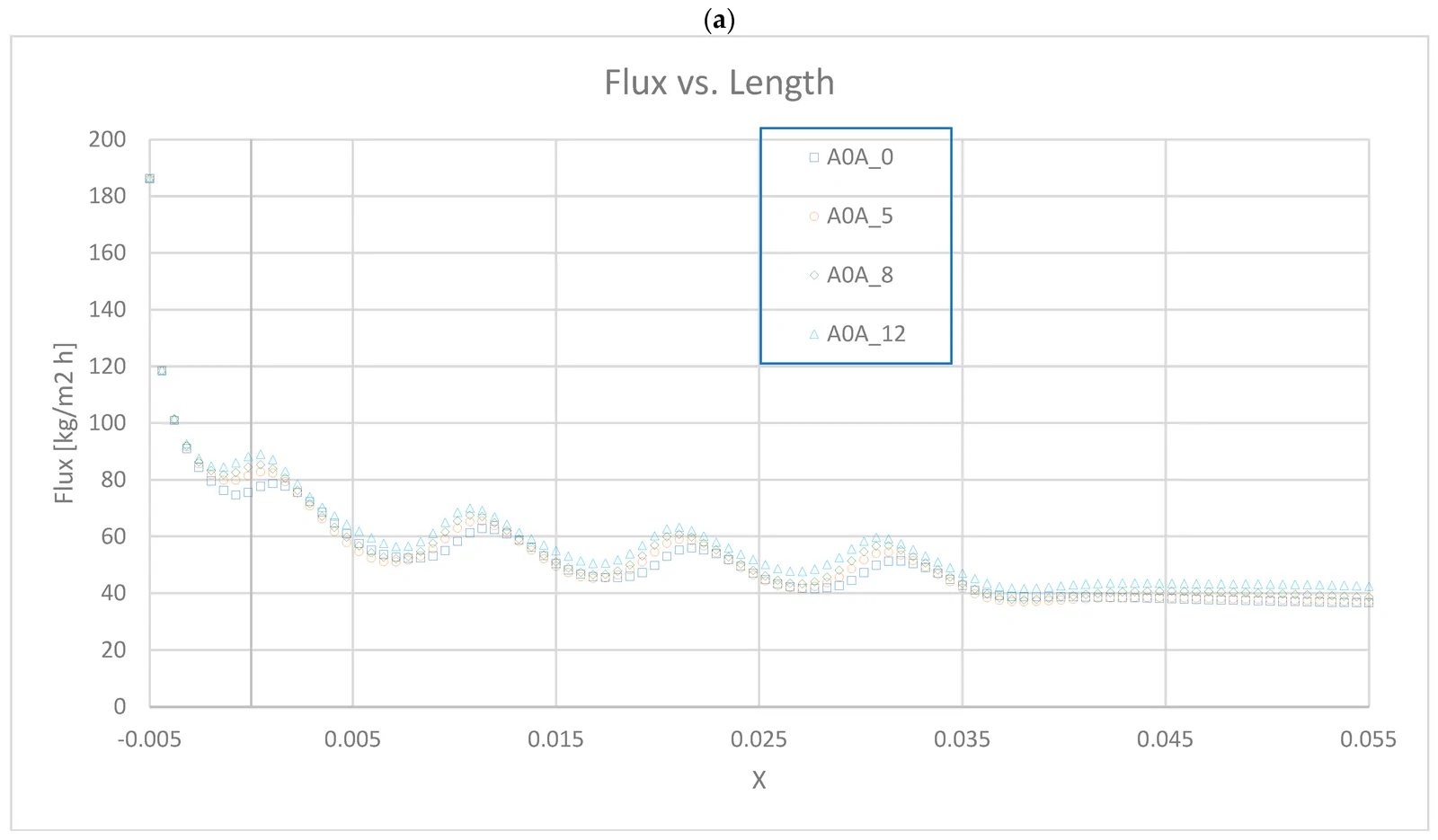

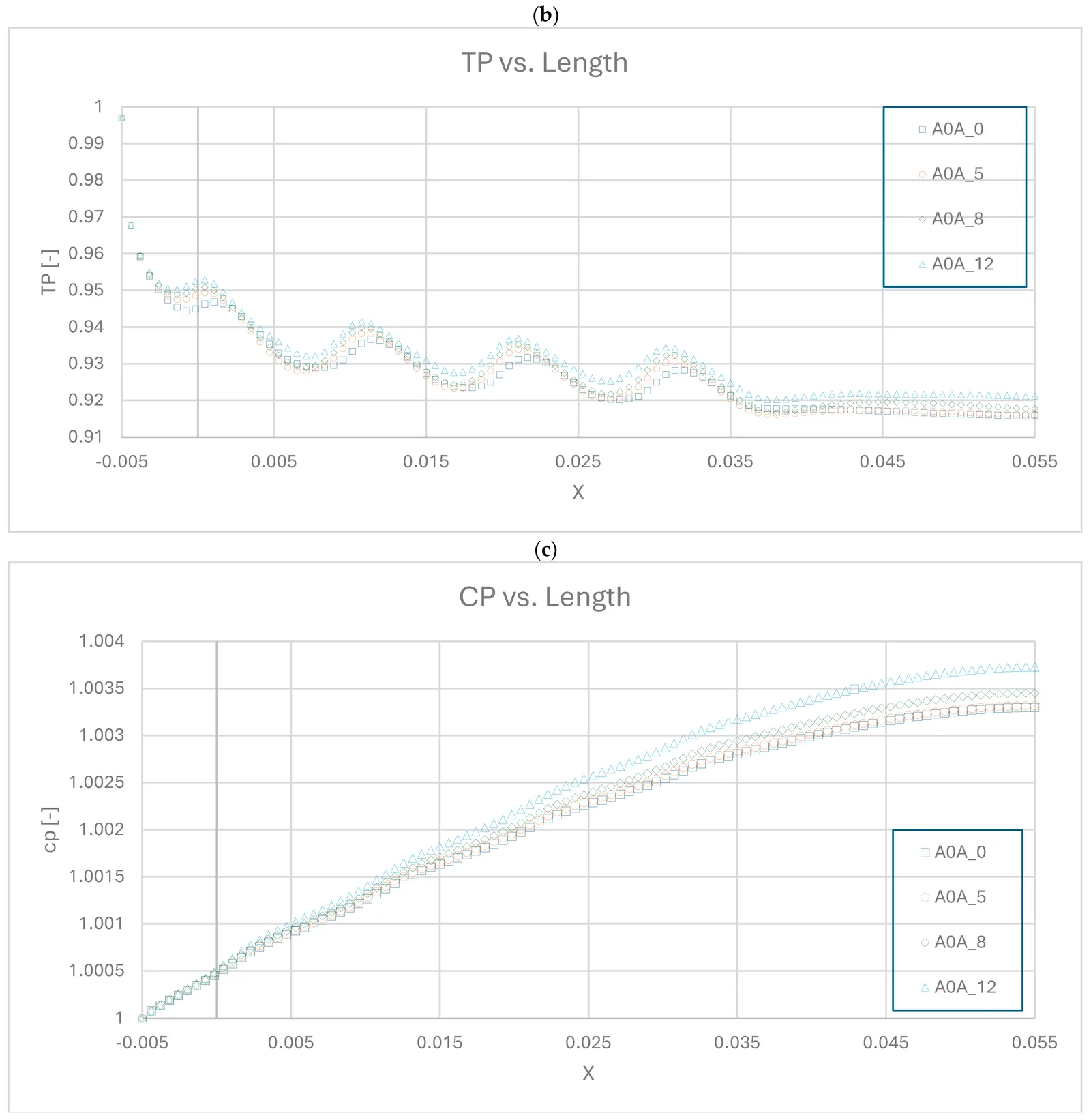
Local profiles of (**a**) permeate flux, (**b**) TP, and (**c**) CP along the membrane surface for the upward orientation at Re = 1000 (AoA = 0° baseline, 5°, 8°, and 12°).

**Figure 6:**
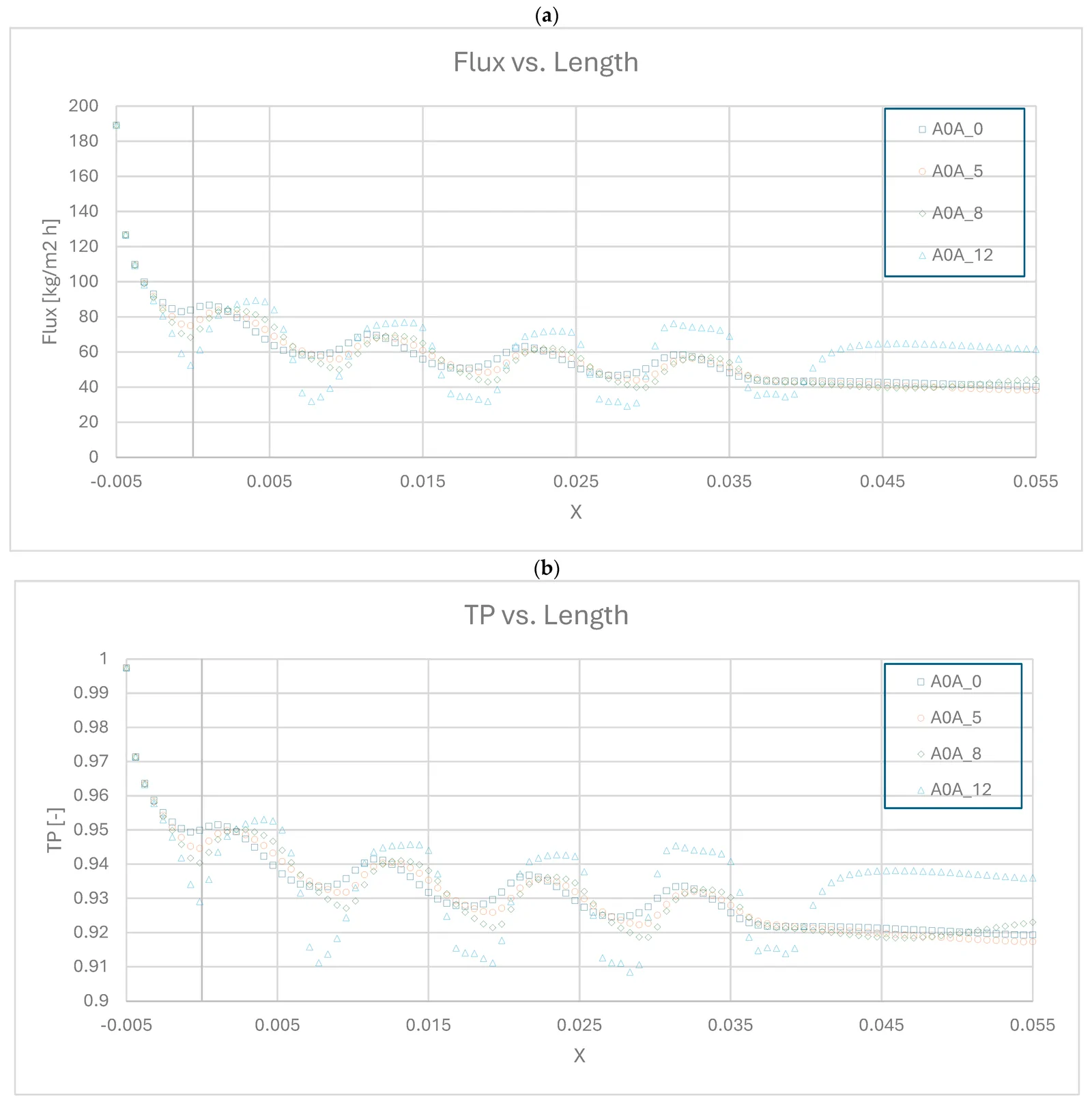

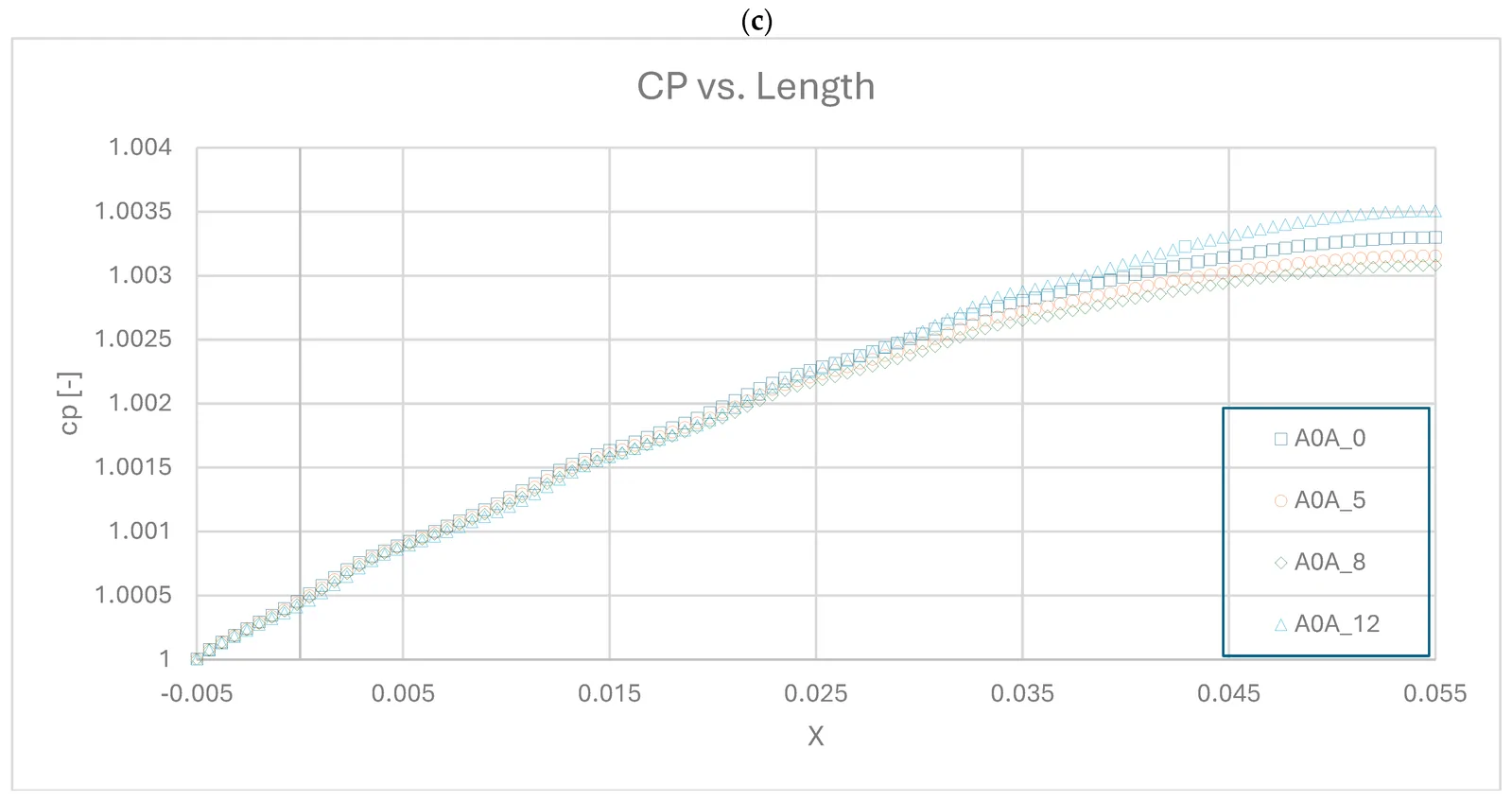
Local profiles of (**a**) permeate flux, (**b**) TP, and (**c**) CP along the membrane surface for the downward orientation at Re = 1500 (AoA = 0° baseline, −5 °, −8 °, and −12 °).

**Figure 7:**
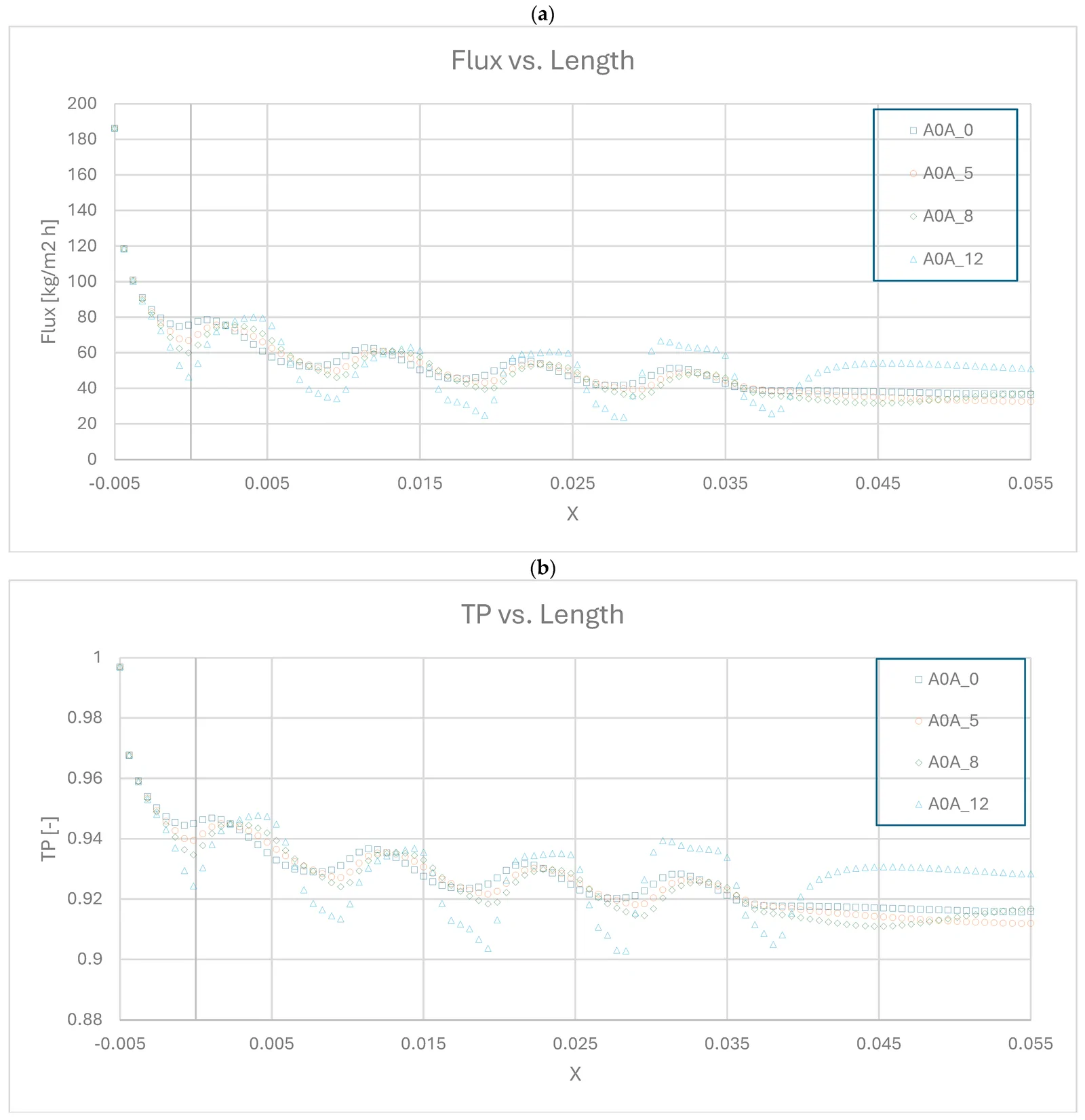

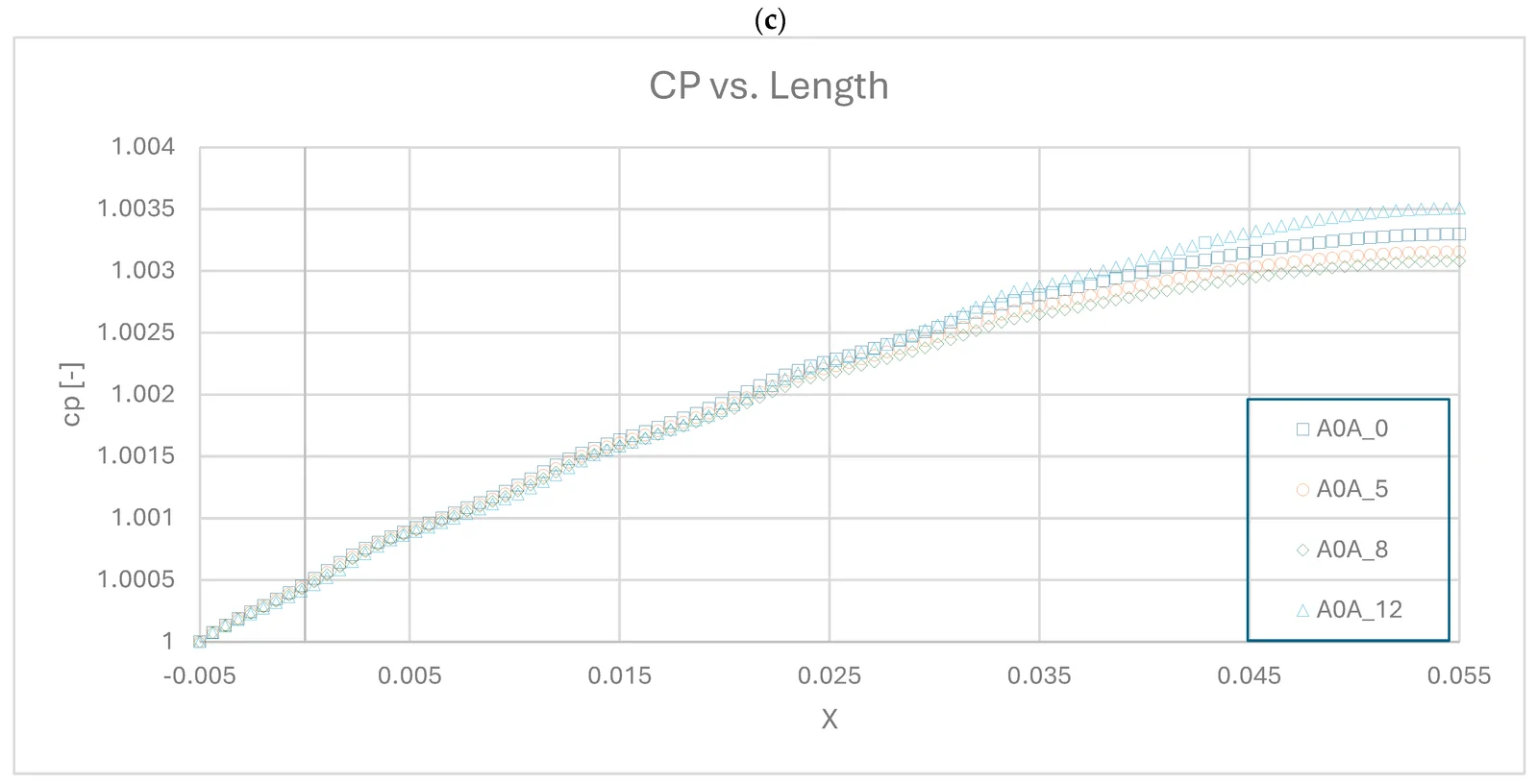
Local profiles of (**a**) permeate flux, (**b**) TP, and (**c**) CP along the membrane surface for the downward orientation at *Re* = 1000 (*AoA* = 0° baseline, −5 °, −8 °, and −12 °).

**Figure 8:**
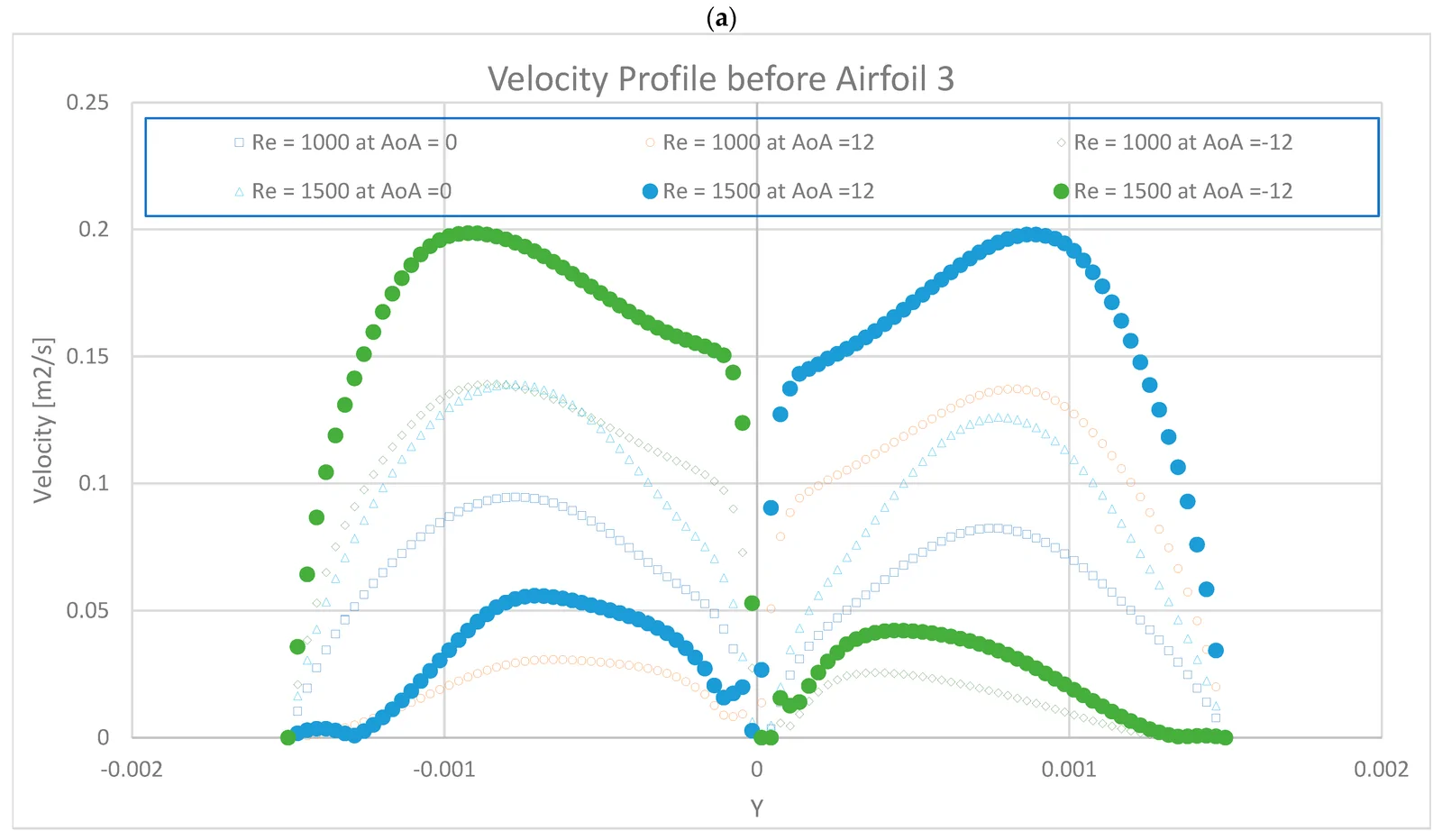

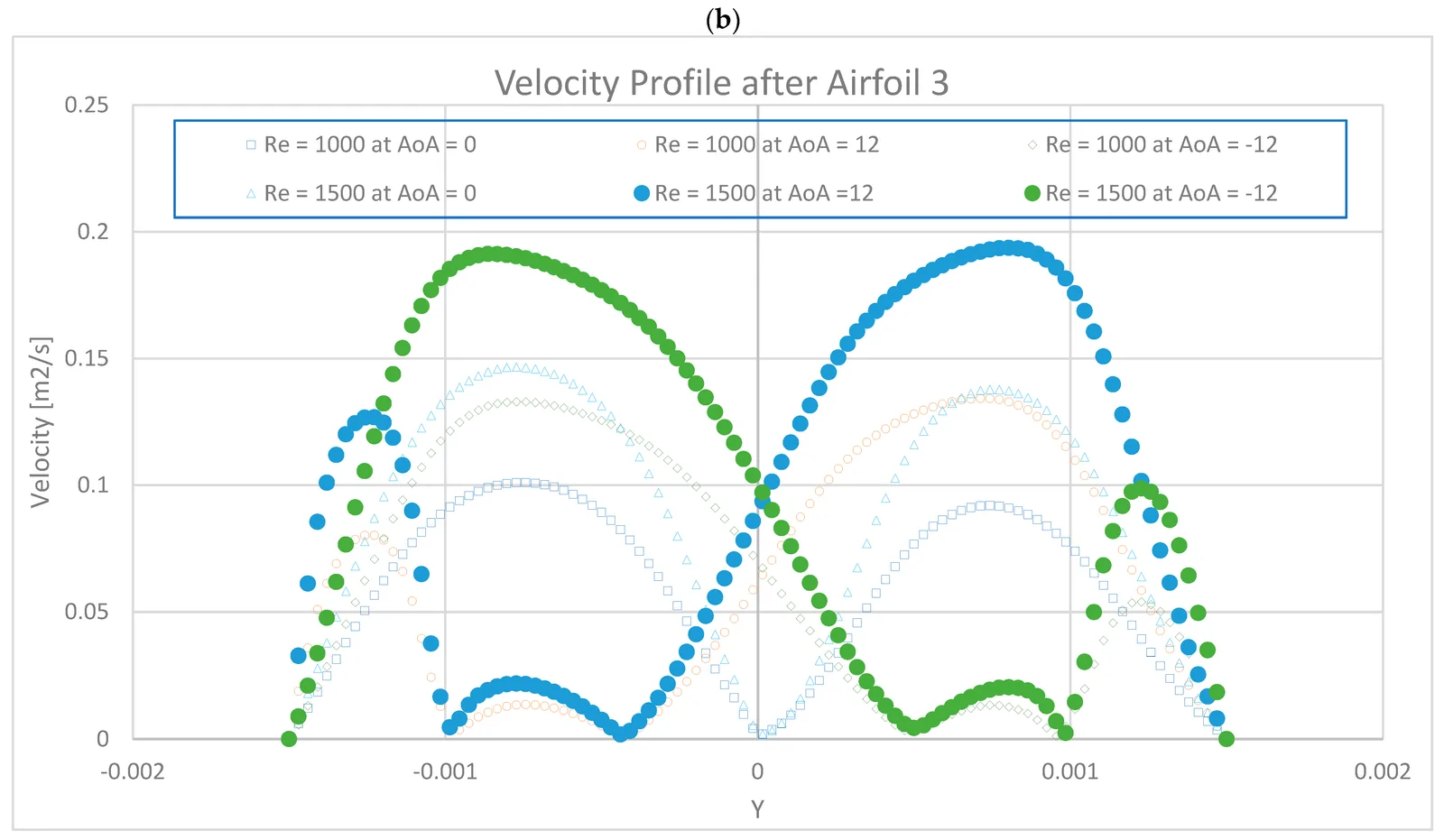
Local velocity field along the Y axis for airfoil 3 at *Re* = 1000 and 1500, and *AoA* = 0,+12,−12. (**a**) Before the airfoil at *x* = 0.02 mm and (**b**) *after the airfoil at* x = 0.025 mm.

**Figure 9:**
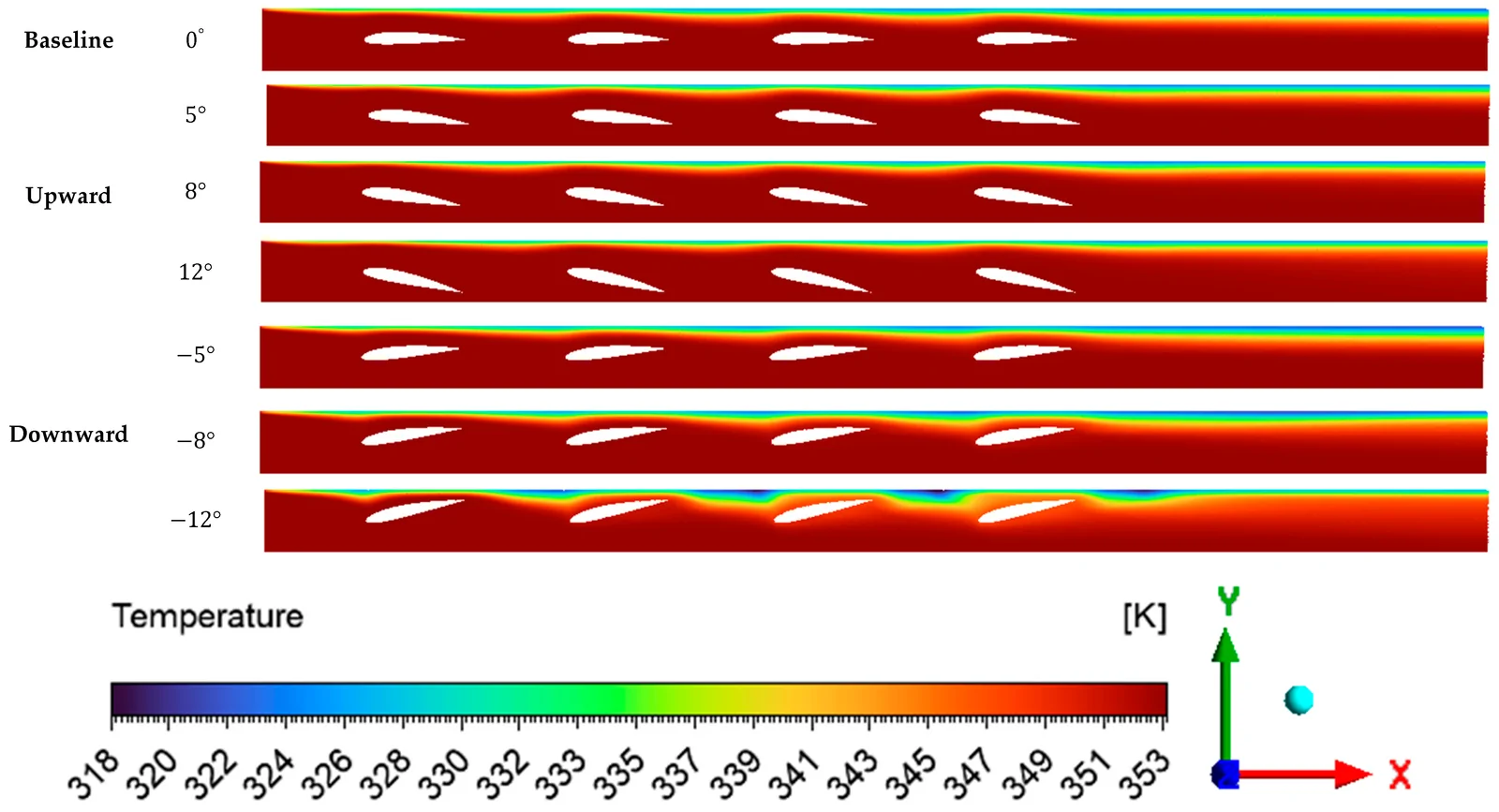
Temperature contour plots at (T_in_ = 80 °C, Re = 1000).

**Figure 10:**
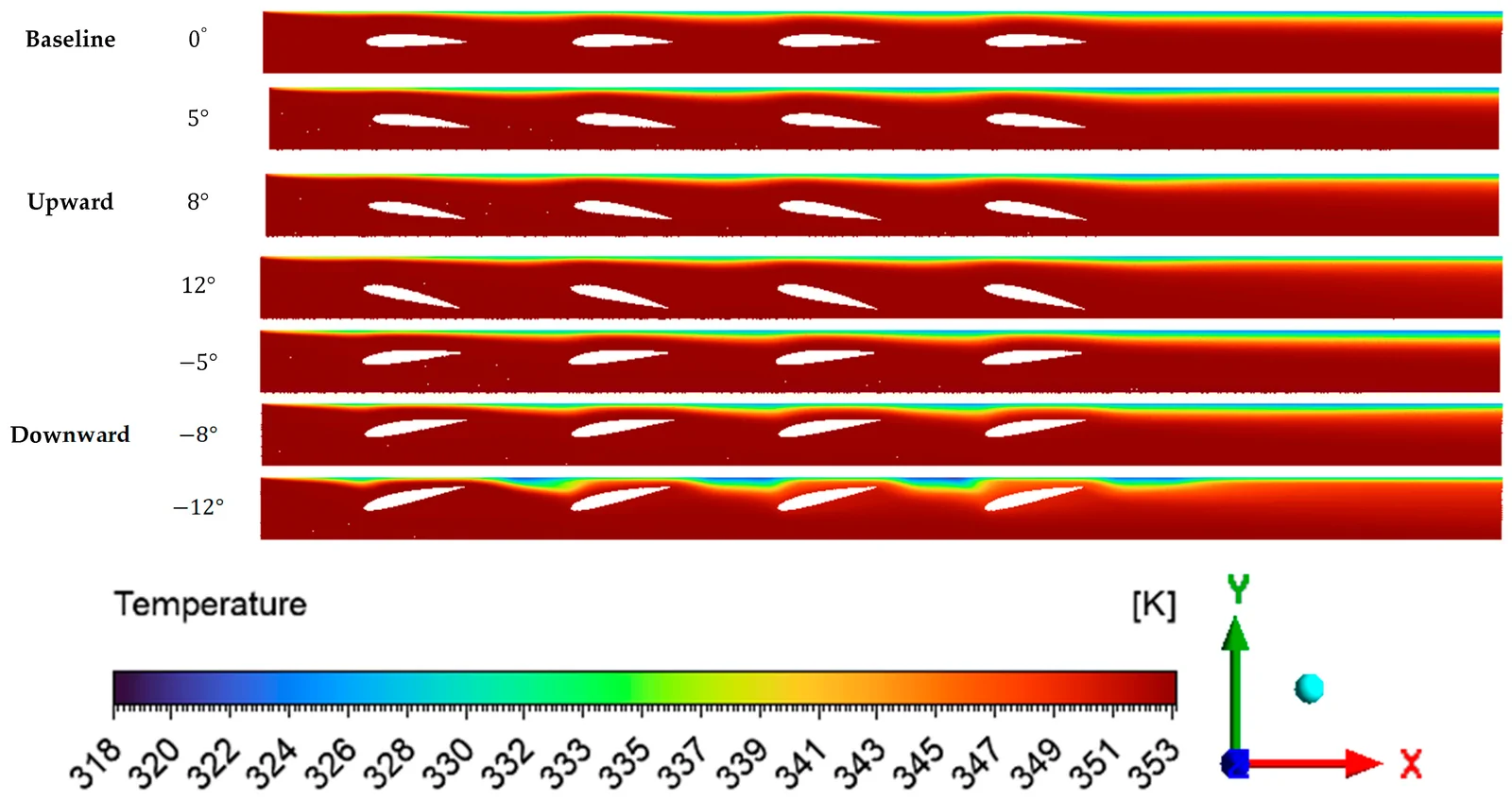
Temperature contour plots at (T_in_ = 80 °C, Re = 1500).

**Figure 11:**
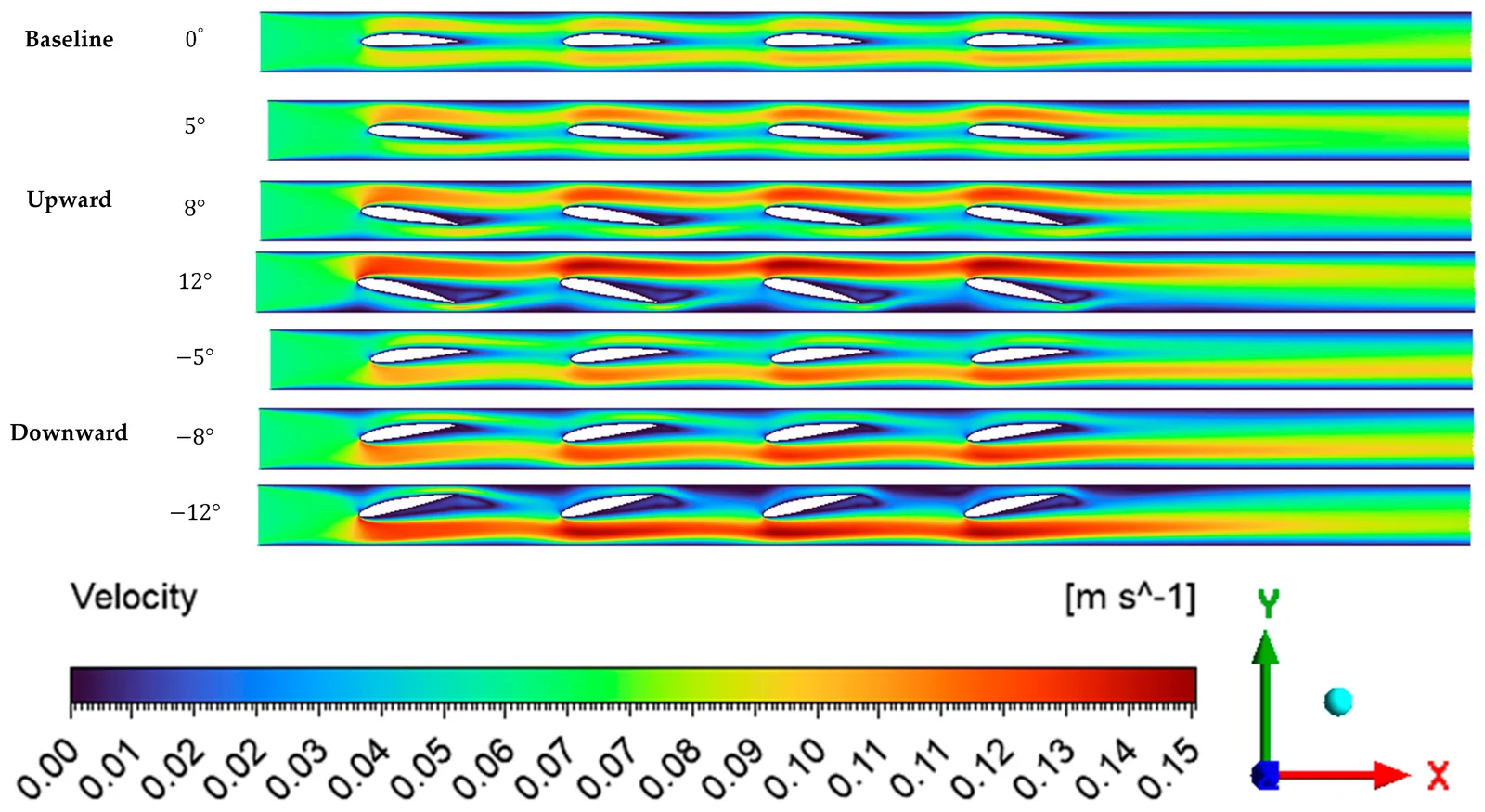
Velocity contour plots at (T_in_ = 80 °C, Re = 1000).

**Figure 12:**
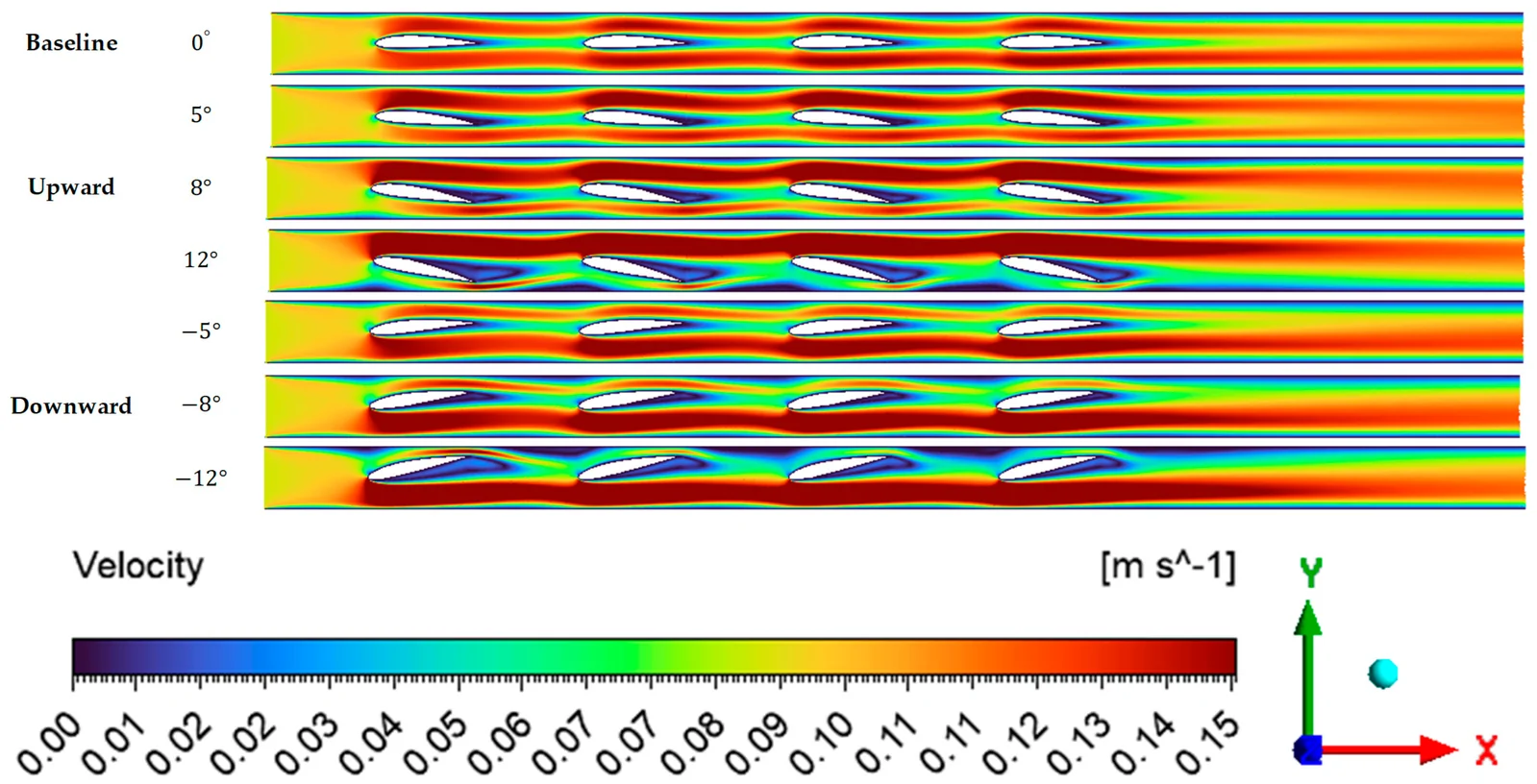
Velocity contour plots at (T_in_ = 80 °C, Re = 1500).

**Figure 13:**
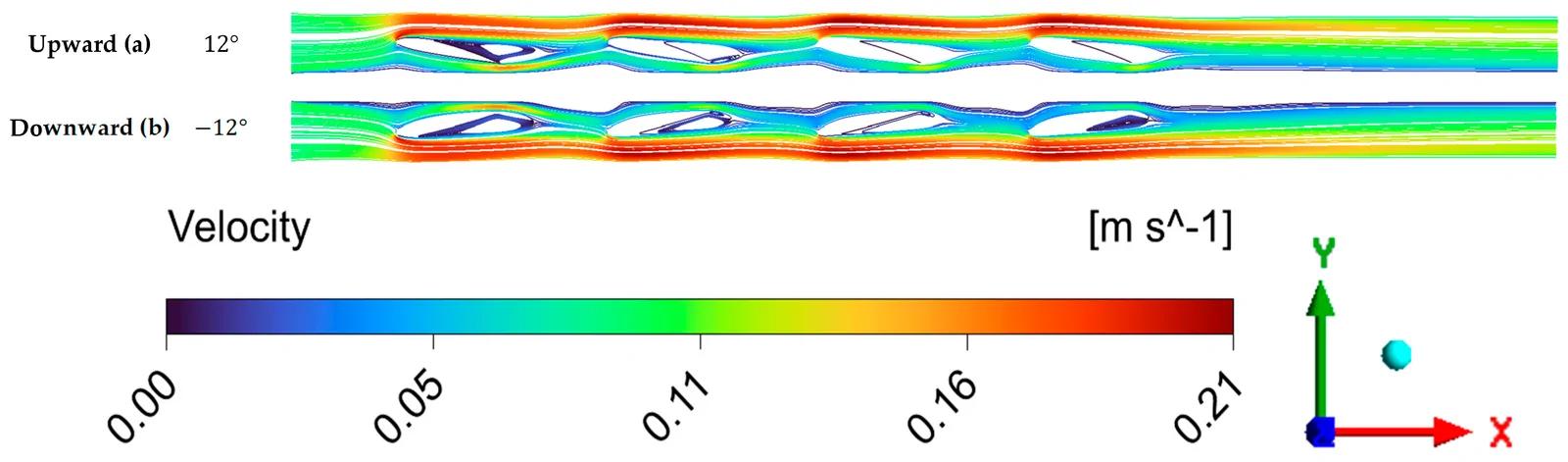
Velocity contours and streamlines around the NACA 2412 airfoil elements at (T_in_ = 80 °C, Re = 1500): (**a**) upward orientation AoA = 12° and (**b**) downward orientation AoA = −12°.

**Table 1:** Summary of the boundary conditions applied to the computational domain.

Boundary	Type	Specification
**Channel inlet**	Velocity inlet	Uniform velocity; *Re* = 1000 and 1500; *T*_in_ = 353.15 K (80 °C); *C*_in_ = 35,000 ppm (35 g/L)
**Channel outlet**	Pressure outlet	Zero-gauge pressure; zero-gradient temperature and concentration
**Bottom wall**	Wall	No slip; adiabatic (∂*T*/∂*y* = 0); impermeable (∂*C*/∂*y* = 0)
**Airfoil surfaces**	Wall	No slip; adiabatic; impermeable
**Membrane interface (top wall)**	Coupled heat and mass sink	Vapor flux *J* = *K*_m_ · (*P_m_*_,*f*_ − *P_m_*_,*p*_); Latent-heat sink *q*_m_ = *J* · Δ*H*_vap_; Complete solute rejection (*J*_s_ = 0); Permeate side held at *P*_v_ = 5 kPa

**Table 2:** Grid independence study results. The selected configuration M3 is bolded.

Mesh	Elements	y^+^ (Airfoil)	y^+^ (Membrane)	Flux [kg/m^2^h]	TPC [−]	CPC [−]
M1	35,000	0.205	0.119	58.12	0.9318	1.0017
M2	70,000	0.143	0.080	57.82	0.9319	1.0017
**M3 ★**	**140,000**	**0.100**	**0.055**	**57.60**	**0.9319**	**1.0017**
M4	240,000	0.074	0.038	57.47	0.9320	1.0017

★ Selected mesh. Variance in flux vs. M4 < 0.5%.

**Table 3:** Validation of the present CFD model against experimental and CFD data from Anqi et al. [78].

T (°C)	Re	Flux Exp. [52] (kg/m^2^h)	Present CFD (kg/m^2^h)	Error vs. Exp. (%)
80	1000	57.36	58.79	2.244
80	200	42.80	35.07	18.05
50	1000	19.70	20.14	2.20
50	602	16.93	17.86	5.21
50	200	11.57	14.07	17.80

**Table 4:** High-level performance summary for optimal configurations at *Re* = 1500.

Configuration	Best Performance Metric	Impact on System
**Upward (*AoA* = 12°)**	Peak flux: 66.0252 kg/m^2^h	Pressure drops 23.5204 Pa (+76.5% vs. baseline)
Downward (*AoA* = −12°)	Moderate flux: 62.1999 kg/m^2^h	Pressure drops 23.3822 Pa (+75.4% vs. baseline)
Airfoil, *α* = 0° (baseline)	Reference flux: 57.6046 kg/m^2^h	Pressure drops 13.3279 Pa (reference)

**Table 5:** Performance metrics for all configurations at *Re* = 1500. Bold rows indicate optimal performance.

Orientation	*AoA* (°)	Flux [kg/m^2^h]	TPC [−]	CPC [−]	ΔP [Pa]
Baseline	0	57.60	0.9319	1.0017	13.3279
**Upward Orientation (α > 0°)**					
Upward	5	59.32	0.9332	1.00175	14.73
Upward	8	61.83	0.9351	1.0018	17.4077
**Upward**	**12**	**66.03**	**0.9381**	**1.0019**	**23.5204**
**Downward Orientation (α < 0°)**					
Downward	−5	56.80	0.9313	1.0017	14.46
Downward	−8	56.45	0.9310	1.0017	16.86
Downward	−12	62.1999	0.9350	1.0018	23.38

Bold row: best-performing upward configuration. TPC and CPC closer to unity indicate less polarization.

**Table 6:** Performance metrics for all configurations at *Re* = 1000.

Orientation	*AoA* (°)	Flux [kg/m^2^h]	TPC [−]	CPC [−]	ΔP [Pa]
Baseline	0	51.74	0.9275	1.0021	2.72
**Upward Orientation (α > 0°)**					
Upward	5	52.38	0.9280	1.0021	8.78201
Upward	8	54.04	0.9293	1.00218	10.0052
**Upward**	**12**	**57.27**	**0.9318**	**1.00234**	**12.6006**
**Downward Orientation (α < 0°)**					
Downward	−5	50.40	0.926365	1.00202	8.68609
Downward	−8	49.43	0.92557	1.00197	9.56425
Downward	−12	53.8808	0.929084	1.0021	12.0521

Note: At *Re* = 1000, the downward orientation causes substantially elevated ΔP relative to upward.

**Table 7:** Merit Number for all configurations.

Re	*Orientation*	*AoA* (°)	Flux Gain	ΔP Increase	Me
1500	Upward	12	+14.6%	+76.5%	0.949
1500	Downward	−12	+8.0%	+75.4%	0.895
1000	Upward	12	+10.7%	+363%	0.664
1000	Downward	−12	+4.1%	+343%	0.634

## Data Availability

All available data generated or analyzed during this study have been included in this published article. No additional datasets were generated or analyzed during the current study.

## Abbreviations

The following abbreviations are used in this manuscript:

**Symbol**	**Description**	**Unit**
**Roman Symbols**		
*c*	Airfoil chord length	m
*C*	Solute (salt) concentration	ppm
*c_p_*	Specific heat capacity	J/(kg K)
*D*	Mass diffusion coefficient of salt	m^2^/s
*H*	Feed channel height	m
Δ*H_vap_*	Latent heat of vaporization of water	J/kg
*J*	Transmembrane permeability flux	kg/(m^2^h)
*J_s_*	Salt rejection flux	kg/(m^2^ s)
*k*	Thermal conductivity	W/(m·K)
*K_m_*	Membrane permeability coefficient	−
*L*	Feed channel length	m
*P*	Pressure	Pa
Δ*P*	Channel pressure drop	Pa
*P*_*m*,*f*_	Partial vapor pressure at the feed-side interface	Pa
*P*_*m*,*p*_	Vacuum pressure on the permeate side	Pa
*P_v_*	Applied vacuum pressure	Pa
*q_m_*	Membrane heat flux (latent)	W/m^2^
*Re*	Reynolds number	−
*T*	Temperature	K
*T_m_*	Local temperature at the membrane interface	K
*u*	Velocity vector	m/s
**Greek Symbols**		
*α*	Angle of attack (*AoA*)	°
*μ*	Dynamic viscosity	Pa s
*ρ*	Fluid density	kg/m^3^
**Acronyms/Subscripts**		
*AoA*	Angle of attack	°
*CFD*	Computational Fluid Dynamics	—
*CPC*	Concentration Polarization Coefficient	−
*MD*	Membrane distillation	—
*TPC*	Temperature Polarization Coefficient	−
*VMD*	Vacuum membrane distillation	—
*P**T**F**E*	Polytetrafluoroethylene	—
*PVDF*	Polyvinylidene fluoride	—

## References

[1] Lawson K.W., Lloyd D.R. (1996). Membrane distillation. I. Module design and performance evaluation using vacuum membrane distillation. J. Membr. Sci..

[2] Alkhudhiri A., Darwish N., Hilal N. (2012). Membrane distillation: A comprehensive review. Desalination.

[3] Abu-Zeid M.A.E.R., Zhang Y., Dong H., Zhang L., Chen H.L., Hou L. (2015). A comprehensive review of vacuum membrane distillation technique. Desalination.

[4] Chiam C.K., Sarbatly R. (2013). Vacuum membrane distillation processes for aqueous solution treatment—A review. Chem. Eng. Process. Process Intensif..

[5] Alklaibi A.M., Lior N. (2004). Membrane-distillation desalination: Status and potential. Desalination.

[6] Curto D., Franzitta V., Guercio A. (2021). A Review of the Water Desalination Technologies. Appl. Sci..

[7] Bandini S., Sarti G.C. (2002). Concentration of must through vacuum membrane distillation. Desalination.

[8] Soni V., Abildskov J., Jonsson G. (2008). Gani, Modeling and analysis of vacuum membrane distillation for the recovery of volatile aroma compounds from black currant juice. J. Membr. Sci..

[9] Al-Asheh S., Banat F., Qtaishat M., El-Khateeb M.Y. (2006). Concentration of sucrose solutions via vacuum membrane distillation. Desalination.

[10] Kast W., Hohenthanner C.R. (2000). Mass transfer within the gas-phase of porous media. Int. J. Heat Mass Transf..

[11] Ding Z., Ma R., Fane A.G. (2003). A new model for mass transfer in direct contact membrane distillation. Desalination.

[12] Baghel R., Upadhyaya S., Singh K., Chaurasia S.P., Gupta A.B., Dohare R.K. (2017). A review on membrane applications and transport mechanisms in vacuum membrane distillation. Rev. Chem. Eng..

[13] Bandini S., Gostoli C., Sarti G.C. (1992). Separation efficiency in vacuum membrane distillation. J. Membr. Sci..

[14] Bandini S., Sarti G.C. (1999). Heat and mass transport resistances in vacuum membrane distillation per drop. AIChE J..

[15] Dao T.D., Mericq J.P., Laborie S. (2013). Cabassud, A new method for permeability measurement of hydrophobic membranes in Vacuum Membrane Distillation process. Water Res..

[16] Liu J., Liu M., Guo H., Zhang W., Xu K., Li B. (2017). Mass transfer in hollow fiber vacuum membrane distillation process based on membrane structure. J. Membr. Sci..

[17] Khayet M., Matsuura T. (2004). Pervaporation and vacuum membrane distillation processes: Modeling and experiments. AIChE J..

[18] Eykens L., Reyns T., De Sitter K., Dotremont C., Pinoy L., Van der Bruggen B. (2016). How to select a membrane distillation configuration? Process conditions and membrane influence unraveled. Desalination.

[19] Martínez-Díez L., Vázquez-González M.I. (1999). Temperature and concentration polarization in membrane distillation of aqueous salt solutions. J. Membr. Sci..

[20] Gryta M., Tomaszewska M. (1998). Heat transport in the membrane distillation process. J. Membr. Sci..

[21] Banat F.A., Simandl J. (1998). Desalination by Membrane Distillation: A Parametric Study. Sep. Sci. Technol..

[22] Qusay F.A., Ibrahim S.S., Khaleel S.R. (2017). Performance of vacuum poly(propylene) membrane distillation (VMD) for saline water desalination. Chem. Eng. Process.-Process Intensif..

[23] Liu J., Li X., Zhang W., Li B., Liu C. (2020). Superhydrophobic-slip surface based heat and mass transfer mechanism in vacuum membrane distillation. J. Membr. Sci..

[24] Julian H., Lian B., Li H., Liu X., Wang Y., Leslie G., Chen V. (2018). Numerical study of CaCO3 scaling in submerged vacuum membrane distillation and crystallization (VMDC). J. Membr. Sci..

[25] Zhang J., Li J.D., Duke M., Xie Z., Gray S. (2010). Performance of asymmetric hollow fibre membranes in membrane distillation under various configurations and vacuum enhancement. J. Membr. Sci..

[26] Li B., Sirkar K.K. (2005). Novel membrane and device for vacuum membrane distillation-based desalination process. J. Membr. Sci..

[27] Tang N., Zhang H., Wang W. (2011). Computational fluid dynamics numerical simulation of vacuum membrane distillation for aqueous NaCl solution. Desalination.

[28] Upadhyaya S., Singh K., Chaurasia S.P., Dohare R.K., Agarwal M. (2015). Mathematical and CFD modeling of vacuum membrane distillation for desalination, Desalin. Water Treat..

[29] Bouguecha S., Chouikh R., Dhahbp M. (2002). Numerical study of the coupled heat and mass transfer in membrane distillation. Desalination.

[30] Baghel R., Kalla S., Upadhyaya S., Chaurasia S.P., Singh K. (2020). CFD modeling of vacuum membrane distillation for removal of Naphthol blue black dye from aqueous solution using COMSOL multiphysics. Chem. Eng. Res. Des..

[31] Chernyshov M.N., Meindersma G.W., de Haan A.B. (2003). Modelling temperature and salt concentration distribution in membrane distillation feed channel. Desalination.

[32] Xu Z., Pan Y., Yu Y. (2009). CFD simulation on membrane distillation of NaCl solution. Front. Chem. Eng. China.

[33] Xu Y., Zhu B.K., Xu Y.Y. (2006). Pilot test of vacuum membrane distillation for seawater desalination on a ship. Desalination.

[34] Yadav A., Singh C.P., Patel R.V., Labhasetwar P.K., Shahi V.K. (2022). Computational fluid dynamics based numerical simulations of heat transfer, fluid flow and mass transfer in vacuum membrane distillation process. Water Supply.

[35] Zuo G., Guan G., Wang R. (2014). Numerical modeling and optimization of vacuum membrane distillation module for low-cost water production. Desalination.

[36] Tahvildari K., Zarabpour A., Ghadiri M., Hemmati A. (2014). Numerical Simulation Studies on Heat and Mass Transfer Using Vacuum Membrane Distillation. Polym. Eng. Sci..

[37] Ghadiri M., Abkhiz V., Parvini M., Marjani A. (2014). Simulation of membrane distillation for purifying water containing 1,1,1-trichloroethane. Chem. Eng. Technol..

[38] Alsaadi A.S., Francis L., Amy G.L., Ghaffour N. (2014). Experimental and theoretical analyses of temperature polarization effect in vacuum membrane distillation. J. Membr. Sci..

[39] Efome J.E., Baghbanzadeh M., Rana D., Matsuura T., Lan C.Q. (2015). Effects of superhydrophobic SiO_2_ nanoparticles on the performance of PVDF flat sheet membranes for vacuum membrane distillation. Desalination.

[40] Garofalo A., Carnevale M.C., Donato L., Drioli E., Alharbi O., Aljlil S.A., Criscuoli A., Algieri C. (2016). Scale-up of MFI zeolite membranes for desalination by vacuum membrane distillation. Desalination.

[41] Liu X., Niu C., Chen X., Wang Z., Cheong K.B. (2025). Experimental investigation on the characteristics of saltwater droplets ice accretion on an NACA0018 airfoil. Appl. Therm. Eng..

[42] El Kadi K., Janajreh I., Hashaikeh R. (2020). Numerical simulation and evaluation of spacer-filled direct contact membrane distillation module. Appl. Water Sci..

[43] Hasani S.M.F., Sowayan A.S., Shakaib M. (2019). The Effect of Spacer Orientations on Temperature Polarization in a Direct Contact Membrane Distillation Process Using 3-d CFD Modeling. Arab. J. Sci. Eng..

[44] Al-Muallem H., Mansour R.B., Antar M.A. (2025). Numerical simulation of the helical strake vacuum membrane distillation. Results Eng..

[45] Loussif N., Orfi J. (2025). Effects of Metal Foam Insertion on the Performance of a Vacuum Membrane Distillation Unit. Membranes.

[46] Khatab M.Z., Abdelsamie M.M., Arafat H.A., Ali M.I.H. (2025). A novel planar module design for improved direct contact membrane distillation: Experimental and computational fluid dynamics insights. Int. J. Thermofluids.

[47] Alwatban A.M., Alhazmi M.A., Aljumaily M.M., Alsalhy Q.F. (2025). Computational fluid dynamics simulations of desalination processes in vacuum membrane distillation, Desalin. Water Treat..

[48] Pang Z., Hou C., Xie S., Wong N.H., Sunarso J., Peng Y. (2022). Optimization of hollow fiber membrane module for vacuum membrane distillation (VMD) via experimental study. Desalination.

[49] Seo J., Kim Y.M., Kim J.H. (2017). Spacer optimization strategy for direct contact membrane distillation: Shapes, configurations, diameters, and numbers of spacer filaments. Desalination.

[50] Tan Y.Z., Ang E.H., Chew J.W. (2019). Metallic spacers to enhance membrane distillation. J. Membr. Sci..

[51] Ni W., Li Y., Zhang G., Du X. (2022). Study of spacer structure on the enhancement of heat and mass transfer in direct contact membrane distillation modules. Desalination.

[52] Thomas N., Kumar M., Palmisano G., Al-Rub R.K.A., Alnuaimi R.Y., Alhseinat E., Rowshan R., Arafat H.A. (2021). Antiscaling 3D printed feed spacers via facile nanoparticle coating for membrane distillation. Water Res..

[53] Albeirutty M., Turkmen N., Al-Sharif S., Bouguecha S., Malik A., Faruki O., Cipollina A., Ciofalo M., Micale G. (2018). An experimental study for the characterization of fluid dynamics and heat transport within the spacer-filled channels of membrane distillation modules. Desalination.

[54] Jeong S., Gu B., Choi G.H., Kim C.B., Jeong S. (2025). CNT spacer-induced cooling crystallisation: A novel approach to mitigate membrane scaling in membrane distillation without chemicals. Clean Water.

[55] Al-juboori R.A., Naji O., Bowtell L., Alpatova A., Soukane S., Ghaffour N. (2021). Power effect of ultrasonically vibrated spacers in air gap membrane distillation: Theoretical and experimental investigations. Sep. Purif. Technol..

[56] Ahmed F.E., Ibrahim Y., Hilal N. (2024). A spacer-based approach for localized Joule heating in membrane distillation. Clean Water.

[57] Jeong Y.H., Jeong S., Jeong S., Gu B. (2024). Helical strip-type spacers for enhanced multi-directional mixing and mitigation of temperature polarisation in membrane distillation—A numerical and experimental investigation. J. Membr. Sci..

[58] Jeong S., Gu B., Choi S., Ahn S.K., Lee J., Lee J., Jeong S. (2023). Engineered multi-scale roughness of carbon nanofiller-embedded 3D printed spacers for membrane distillation. Water Res..

[59] Ahmed F., El Kadi K., Hashaikeh R., Janajreh I. (2017). Low Energy Membrane Distillation: A Numerical Study on The Role of Conductive Spacers. Energy Procedia.

[60] Li Q., Lian B., Zhong W., Omar A., Razmjou A., Dai P., Guan J., Leslie G., Taylor R.A. (2020). Improving the performance of vacuum membrane distillation using a 3D-printed helical baffle and a superhydrophobic nanocomposite membrane. Sep. Purif. Technol..

[61] Chang H., Hsu J.A., Chang C.L., Ho C.D., Cheng T.W. (2017). Simulation study of transfer characteristics for spacer-filled membrane distillation desalination modules. Appl. Energy.

[62] Karimi S., Morciano M., Turmo C.P., Piredda M.M., Asinari P., Gloth O., Fasano M. (2026). Mitigating polarization in flat-sheet membrane distillation through CFD-driven spacer design. Sci. Rep..

[63] Kim Y.D., Francis L., Lee J.G., Ham M.G., Ghaffour N. (2018). Effect of non-woven net spacer on a direct contact membrane distillation performance: Experimental and theoretical studies. J. Membr. Sci..

[64] Alwatban A.M., Alshwairekh A.M., Alqsair U.F., Alghafis A.A., Oztekin A. (2019). Performance improvements by embedded spacer in direct contact membrane distillation—Computational study. Desalination.

[65] Hagedorn A., Fieg G., Winter D., Koschikowski J., Grabowski A., Mann T. (2017). Membrane and spacer evaluation with respect to future module design in membrane distillation. Desalination.

[66] Zhang Z., Zhang N., Xiang B., Yuan Y., Phelan P.E. (2023). Numerical investigation on mass and heat transfer performance for novel vacuum membrane distillation modules enhanced by semicircular spacers. Int. J. Therm. Sci..

[67] Alshwairekh A.M., Alghafis A.A., Alwatban A.M., Alqsair U.F., Oztekin A. (2019). The effects of membrane and channel corrugations in forward osmosis membrane modules—Numerical analyses. Desalination.

[68] Zhou Z., Ladner D.A. (2022). Computational modeling of discrete-object feed spacers attached directly onto reverse osmosis membranes for enhanced module packing capacity and improved hydrodynamics. Sep. Purif. Technol..

[69] Naidu G., Shim W.G., Jeong S., Choi Y.K., Ghaffour N., Vigneswaran S. (2017). Transport phenomena and fouling in vacuum enhanced direct contact membrane distillation: Experimental and modelling. Sep. Purif. Technol..

[70] EL-Bourawi M.S., Khayet M., Ma R., Ding Z., Li Z., Zhang X. (2007). Application of vacuum membrane distillation for ammonia removal. J. Membr. Sci..

[71] Abdallah H., Moustafa A.F., AlAnezi A.A.H., El-Sayed H.E.M. (2014). Performance of a newly developed titanium oxide nanotubes/polyethersulfone blend membrane for water desalination using vacuum membrane distillation. Desalination.

[72] Qamar A., Kerdi S., Vrouwenvelder J.S., Ghaffour N. (2023). Airfoil-shaped filament feed spacer for improved filtration performance in water treatment. Sci. Rep..

[73] Zhang F., Gu C., Cheng Y., Chen R., Wang Z., Cui Z. (2026). An integrated module with synergistic heating and turbulence promotion for enhanced vacuum membrane distillation. J. Membr. Sci..

[74] Kotb M.A., Elboghdadi M.M., Antar M.A., Abido M.A., Khalifa A.E. (2026). Wind-powered multistage vacuum membrane distillation for sustainable water desalination. Appl. Therm. Eng..

[75] Abdallah S.B., Khitouni M., Alminderej F.M., Frikha N., Gabsi S. (2026). Performance evaluation and fouling behavior of a submerged vacuum membrane distillation system for desalination. Mater. Today Commun..

[76] NACA NACA 2412, UIUC Appl. Aerodyn. Gr. (n.d.). http://airfoiltools.com/airfoil/details?airfoil=naca2412-il.

[77] Celik I., Karatekin O. (1997). Numerical Experiments on Application of Richardson Extrapolation with Nonuniform Grids. J. Fluids Eng..

[78] Anqi A.E., Usta M., Krysko R., Lee J.G., Ghaffour N., Oztekin A. (2020). Numerical study of desalination by vacuum membrane distillation—Transient three-dimensional analysis. J. Membr. Sci..

